# Shorter Peptide Nucleic Acid Probes Improve Affibody-Mediated Peptide Nucleic Acid-Based Pretargeting

**DOI:** 10.1021/acsptsci.4c00106

**Published:** 2024-04-29

**Authors:** Kristina Westerlund, Maryam Oroujeni, Maxime Gestin, Jacob Clinton, Alia Hani Rosly, Hanna Tano, Anzhelika Vorobyeva, Anna Orlova, Amelie Eriksson Karlström, Vladimir Tolmachev

**Affiliations:** † Department of Protein Science, School of Engineering Sciences in Chemistry, Biotechnology and Health, KTH Royal Institute of Technology, Stockholm 106 91, Sweden; ‡ Department of Immunology, Genetics and Pathology, Uppsala University, Uppsala 751 23, Sweden; § Affibody AB, Solna 171 65, Sweden; ∥ Department of Medicinal Chemistry, Uppsala University, Uppsala 751 23, Sweden

**Keywords:** affibody, pretargeting, peptide nucleic acid, radiotherapy

## Abstract

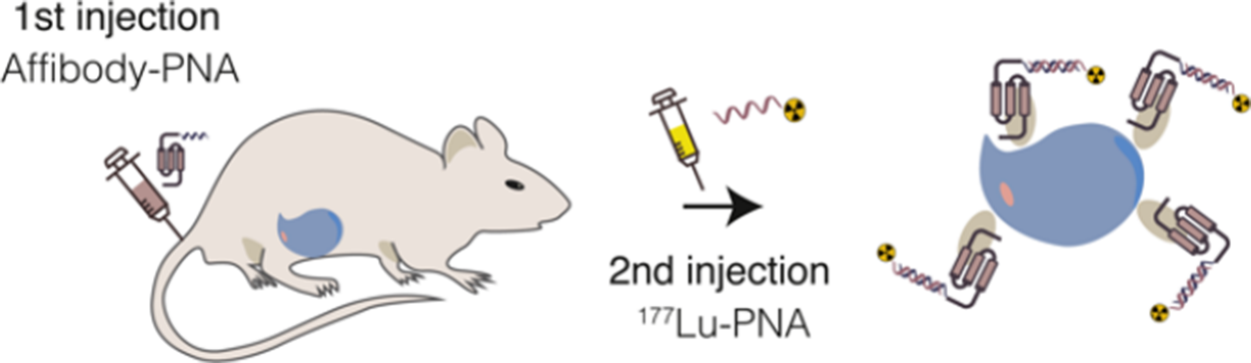

Affibody-mediated PNA-based pretargeting shows promise for HER2-expressing tumor radiotherapy. In our recent study, a 15-mer Z_HER2:342_-HP15 affibody-PNA conjugate, in combination with a shorter 9-mer [^177^Lu]Lu-HP16 effector probe, emerged as the most effective pretargeting strategy. It offered a superior tumor-to-kidney uptake ratio and more efficient tumor targeting compared to longer radiolabeled effector probes containing 12 or 15 complementary PNA bases. To enhance the production efficiency of our pretargeting system, we here introduce even shorter 6-, 7-, and 8-mer secondary probes, designated as HP19, HP21, and HP20, respectively. We also explore the replacement of the original 15-mer Z-HP15 primary probe with shorter 12-mer Z-HP12 and 9-mer Z-HP9 alternatives. This extended panel of shorter PNA-based probes was synthesized using automated microwave-assisted methods and biophysically screened in vitro to identify shorter probe combinations with the most effective binding properties. In a mouse xenograft model, we evaluated the biodistribution of these probes, comparing them to the Z-HP15:[^177^Lu]Lu-HP16 combination. Tumor-to-kidney ratios at 4 and 144 h postinjection of the secondary probe showed no significant differences among the Z-HP9:[^177^Lu]Lu-HP16, Z-HP9:[^177^Lu]Lu-HP20, and the Z-HP15:[^177^Lu]Lu-HP16 pairs. Importantly, tumor uptake significantly exceeded, by several hundred-fold, that of most normal tissues, with kidney uptake being the critical organ for radiation therapy. This suggests that using a shorter 9-mer primary probe, Z-HP9, in combination with 9-mer HP16 or 8-mer HP20 secondary probes effectively targets tumors while minimizing the dose-limiting kidney uptake of radionuclide. In conclusion, the Z-HP9:HP16 and Z-HP9:HP20 probe combinations offer good prospects for both cost-effective production and efficient in vivo pretargeting of HER2-expressing tumors.

Monoclonal antibodies (mAbs) have a history of successful use as targeting tools in radioimmunotherapy for hematological malignancies.^1^ However, when applied to solid tumors, targeting agents based on full-length antibodies have faced clinical challenges. Issues such as limited penetration into the target tissue, slow passage through blood vessel walls, and extended circulation times for radiolabeled mAbs have resulted in inadequate radiation doses reaching solid tumors. This, in turn, has led to unintended radiation exposure of healthy tissues, notably the radiation-sensitive bone marrow.^2,3^ Engineered scaffold proteins (ESPs) offer a promising alternative for targeted radionuclide therapy and imaging. These nonimmunoglobulin binding proteins are typically much smaller than mAbs, providing several advantages, including enhanced extravasation and improved tissue penetration, and can be engineered to bind specifically and with high affinity to cancer-related molecules.^4^

One well-studied ESP for radionuclide molecular imaging is the affibody molecule, a compact 58-amino acid, three-helix bundle protein with a molecular weight of 7–8 kDa.^5^ Affibody-based imaging probes have shown promise in preclinical studies targeting a range of tumor-associated proteins, including human epidermal growth factor receptor 2 (HER2), human epidermal growth factor receptor 3 (HER3), insulin-like growth factor 1 receptor (IGF-1R), programmed death-ligand 1 (PD-L1), and carbonic anhydrase IX (CAIX).^6^ Selected affibody molecules exhibit high affinities (in the subnanomolar range) for their target proteins, a critical factor for achieving significant accumulation and retention in tumors. Additionally, their rapid binding kinetics and efficient blood clearance make affibody molecules a promising scaffold protein type for the development of radionuclide imaging agents.^7^

One example of a promising affibody molecule is Z_HER2:342_, which exhibits high-affinity binding to the HER2 receptor (*K*_D_ = 22 pM^8^). Preclinical studies have demonstrated its excellent targeting property for HER2-expressing cells.^9−11^ Another noteworthy development is [^68^Ga]Ga-ABY-025, a ^68^Ga-labeled derivative of Z_HER2:342_, which offers increased stability and hydrophilicity. Currently, it is undergoing phase 2 trials for imaging breast and gastroesophageal cancers.^12,13^

However, the direct radiolabeling of affibody molecules for radiotherapy has faced challenges due to efficient renal reabsorption and the accumulation of cytotoxic radionuclides in the kidneys. In some cases, renal retention of residualizing radiometals can exceed tumor uptake by 10–20-fold, potentially leading to dose-limiting kidney toxicity.^14−16^

One solution to this issue is the implementation of a pretargeting approach. In this method, an unlabeled tumor-targeting agent is administered first and allowed to bind to the tumor site and clear from nontargeted organs (see schematic Figure 1). Subsequently, a cytotoxic radiolabeled secondary agent is injected. The secondary agent is designed to interact with the primary agent at the tumor site with high affinity and specificity and to have fast in vivo clearance.

**Figure 1:**
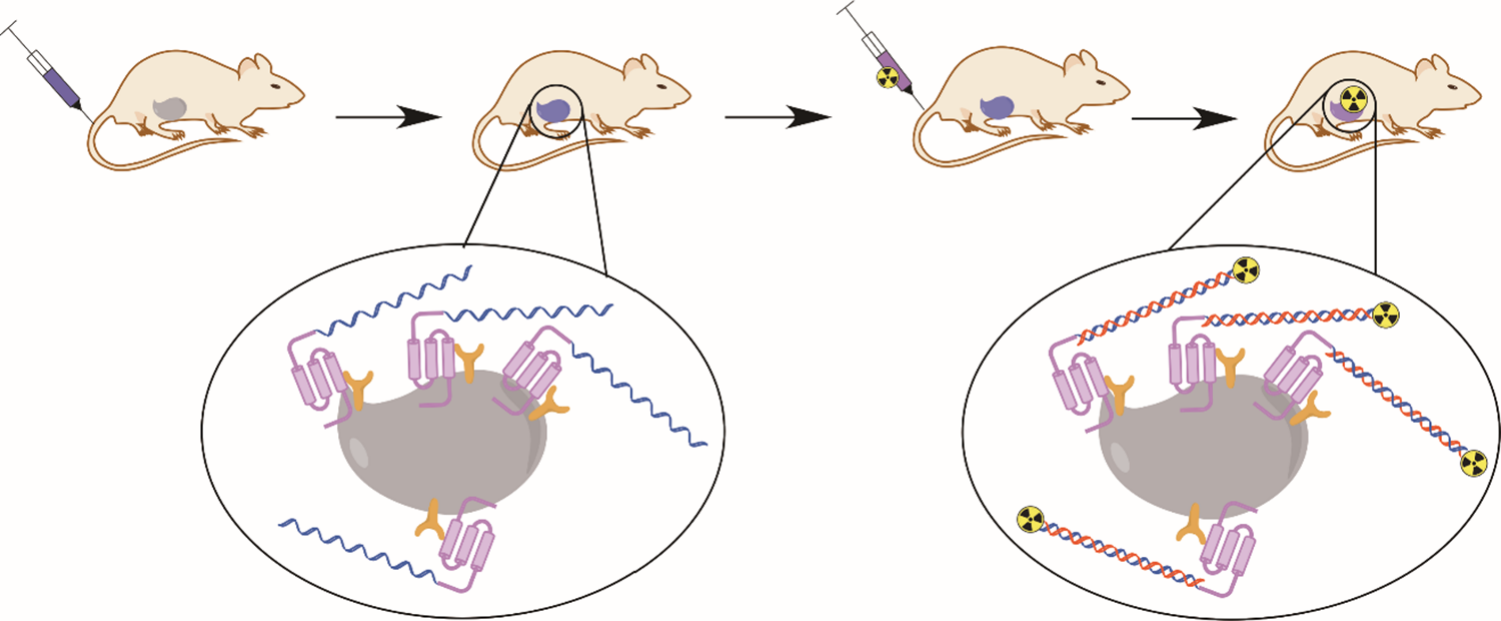
PNA-based affibody-mediated tumor radiotherapy pretargeting strategy in mice. The initial step involves injecting the tumor-targeting primary agent, in this case an affibody-PNA conjugate, which subsequently binds to the tumor tissue. Following clearance from the rest of the body, the radiolabeled secondary agent, a complementary radiolabeled PNA-based probe, is administered and binds to the PNA probe of the primary agent.

All pretargeting methods used to date can be divided into two main categories based on the mode of interaction between the tumor-targeting agent and the radiolabeled effector probe: (I) those that are based on covalent bond formation and bioorthogonal click chemistry in vivo, and (II) those that rely on noncovalent high-affinity binding. The latter group includes methods based on avidin/streptavidin binding to biotin, bispecific antibodies, methods based on oligonucleotide hybridization, and on supramolecular host–guest complexation.^17,18^

Our research group has previously explored affibody-mediated pretargeting using two different approaches: one based on a bioorthogonal cycloaddition reaction between trans-cyclooctene and tetrazine^19^ and the other on peptide nucleic acid (PNA) duplex formation in vivo.^20,21^ Affibody molecules are particularly well-suited for pretargeting applications because they tend to have slow internalization kinetics after binding to tumor cells and are thus available on the cell surface for binding to the effector probe.^16^ Both of these pretargeting methods substantially improved the tumor-to-kidney absorbed dose ratios, but among these approaches, PNA-mediated pretargeting, which relies on synthetic oligonucleotides that hybridize in vivo, demonstrated the highest radionuclide accumulation in tumors, a reduced renal uptake, and was found to be the most efficient.

PNA is a synthetic DNA analogue with a charge neutral peptide-like backbone and can be easily synthesized using standard solid-phase synthesis (SPS) methods and using commercially available building blocks. PNAs can hybridize, following Watson–Crick base pairing rules, to complementary oligonucleotides with higher affinity and specificity than their natural counterparts.^22^ Initially developed for antisense therapy applications, PNAs possess several advantageous properties: they are chemically and thermally stable, resistant to enzymatic hydrolysis, nonimmunogenic, nontoxic, and highly stable in human serum.^23^ However, unmodified PNA oligomers display a limited cellular permeability,^24^ and PNAs have a short serum half-life in vivo due to rapid kidney excretion of unmetabolized PNA through urine.^25^ The reduced cellular uptake of PNA oligomers has posed challenges for intracellular applications. Still, it can prove beneficial in pretargeted therapeutic applications, where fast internalization kinetics and slow blood clearance of the radiolabeled effector probe are undesirable and potentially dose-limiting.

In a preclinical therapy study using the [^177^Lu]Lu-HP2 15-mer effector probe, affibody-mediated pretargeting led to a significant increase in median survival among treated mice (66 days for pretargeted mice compared to 32 days for mice receiving [^177^Lu]Lu-HP2 only).^26^ Another preclinical therapy study employed a combination of affibody-based PNA-mediated pretargeting and the mAb trastuzumab, resulting in a notable increase in mouse survival when compared to monotherapies alone. Importantly, no observable side effects or toxicities were reported.^27^

However, to achieve a curative treatment, further enhancements to the PNA-based pretargeting approach were considered necessary. Given that the tumor uptake of HP2 was several hundred-fold higher than in most other tissues, the kidneys were recognized as the dose-limiting organ. Thus, the challenge lies in finding a way to reduce kidney uptake while still maintaining a high level of uptake in the tumor. One parameter that can significantly influence kidney uptake is the length of the radiolabeled oligonucleotide-based effector probe. Leonidova et al. observed that increasing the PNA chain from a 12-mer to a 17-mer in their pretargeting system raised kidney uptake. However, the ^99m^Tc-labeled 12-mer probe cleared very rapidly from the blood, and the longer 17-mer probe, with an enhanced blood availability, was chosen for further in vivo pretargeting development.^28^

In our recent study,^29^ we introduced a second-generation of pretargeting probes featuring a common 15-mer affibody-PNA conjugate, Z_HER2:343_-HP15, and three complementary secondary probes of varying lengths: a 9-mer (HP16), a 12-mer (HP17), and a 15-mer (HP18). Among these, the Z_HER2:342_-HP15:[^177^Lu]Lu-HP16 combination, with the shortest 9-mer secondary probe, emerged as the most effective pretargeting probe pair in a HER2 xenograft mouse model. This combination achieved a 2-fold higher tumor-to-kidney ratio compared to both the 15-mer Z_HER2:342_-HP15:[^177^Lu]Lu-HP18 probe pair and the first-generation 15-mer Z_HER2:342_-HP1:[^177^Lu]Lu-HP2 probes.^29^ Notably, the kidney uptake for [^177^Lu]Lu-HP16 (6 ± 1% ID/g) was significantly lower than that of [^177^Lu]Lu-HP18 (12 ± 2% ID/g) and [^177^Lu]Lu-HP2 (10 ± 2% ID/g), while the tumor uptake values ranged from 19 to 24% ID/g, with no significant differences observed between the different secondary probes.

The transition to shorter PNA-based probes also provides several production benefits. Generally, the reduction in the number of synthesis steps in SPS results in faster production, decreased reagent requirements, and reduced solvent consumption. A streamlined synthesis process can also result in improved crude purity, simplifying the often time consuming and costly downstream purification. The production of PNA oligomers is additionally complicated by their bulky nucleotide side chains and sterically hindered protecting groups. In addition, PNA monomers tend to have poor solubility, which can lead to inefficient couplings, and the growing PNA chain has a tendency to self-aggregate on the solid support during synthesis.^30^ Furthermore, solubility and aggregation issues might persist even after synthesis, affecting their practical use in common biological buffers.^31^ These challenges become more significant with longer PNA chains, further highlighting the benefits of using shorter PNA-based probes.

The primary PNA-based probe can be shortened in an optimization procedure similar to the secondary probes. However, it is crucial to be aware of the potential risks involved in modifying the affibody-conjugated primary agent. Changes could compromise the affinity and specificity of the affibody-PNA conjugate for their cancer-associated targets, as well as the affinity for the effector probe. Additionally, even minor structural alterations in the tumor-targeting agent can have a significant impact on the biodistribution profile, particularly in the kidneys, as evidenced by several studies.^32−34^

The primary objective of this study was to identify combinations of PNA-based pretargeting probes that combined cost-effective production with a favorable tumor-to-kidney uptake ratio in vivo. To achieve this goal, we introduced an expanded range of second-generation PNA-based probes for affibody-mediated pretargeting. This extended set of probes included three new primary probes: HP12 (12-mer), HP9 (9-mer), and HP6 (6-mer), as well as three new secondary probes: HP20 (8-mer), HP21 (7-mer), and HP19 (6-mer) (refer to Figure 2). These probes were synthesized using microwave heating on an automated peptide synthesizer and were characterized by their shorter PNA oligomer length compared to previous versions. Utilizing microwave heating, in combination with coupling agents, which are particularly suitable for the synthesis of aggregation-prone peptide sequences, has previously proven to be beneficial for PNA synthesis, resulting in improved yields and higher purity of crude products.^35^ All new primary probes featured an N-terminal GGG motif for Sortase A-mediated conjugation to the C-terminus of the anti-HER2 Z_HER2:342_ affibody molecule. Additionally, both the primary and the secondary probes were equipped with a versatile chelator, 1,4,7,10-tetraazacyclododecane-1,4,7,10-tetraacetic acid (DOTA), for radiometal complexation with, for example, ^68^Ga, ^111^In, or ^177^Lu.

**Figure 2:**
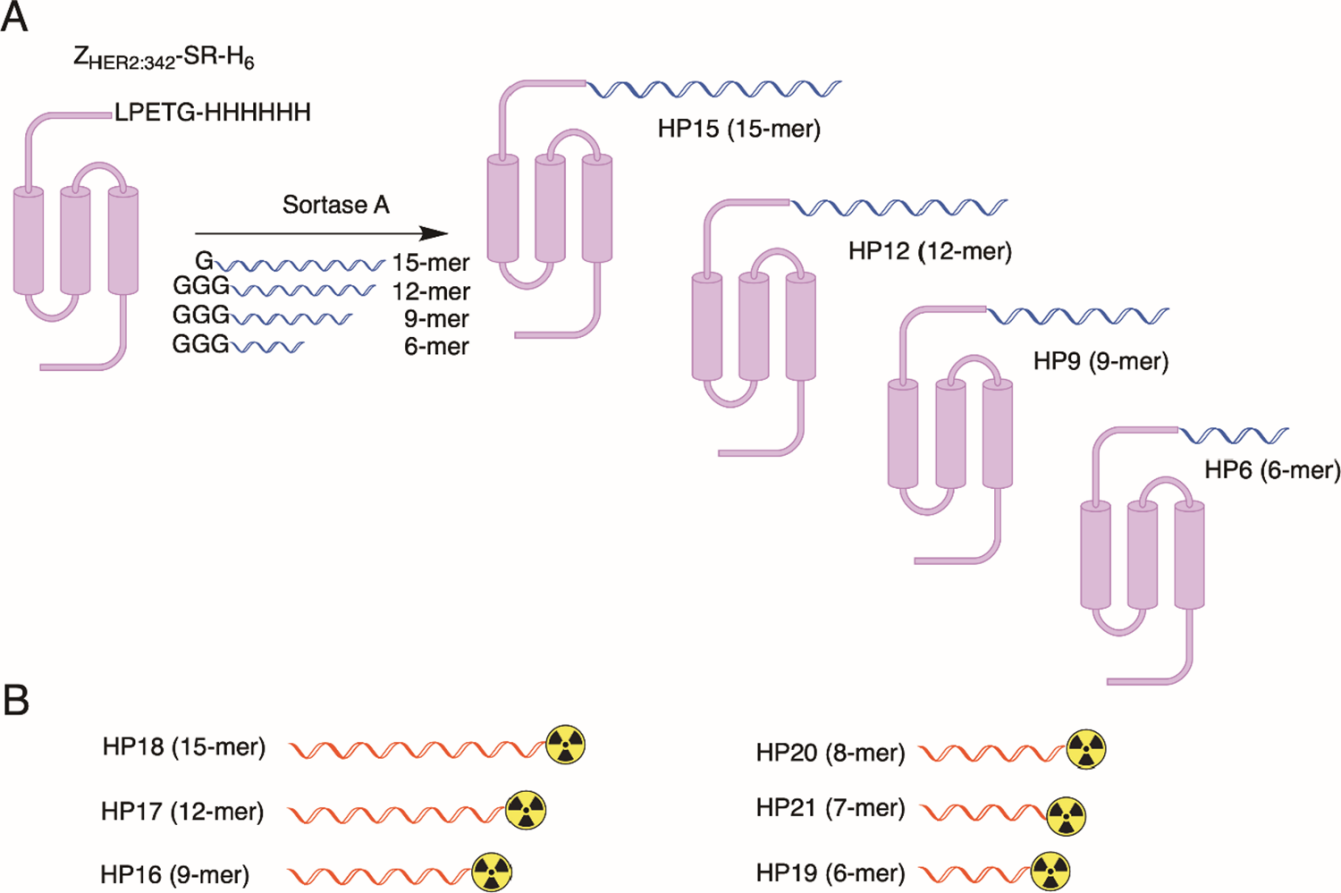
Overview of the various PNA probes and affibody-PNA conjugates in this study. (A) The four primary PNA probes, HP15 (15-mer), HP12 (12-mer), HP9 (9-mer), and HP6 (6-mer), are linked to the affibody Z_HER2:342_ via Sortase A-mediated conjugation. (B) The six secondary PNA probes, HP18 (15-mer), HP17 (12-mer), HP16 (9-mer), HP20 (8-mer), HP21 (7-mer), and HP19 (6-mer), each contain a versatile radiometal chelator, 1,4,7,10-tetraazacyclododecane-1,4,7,10-tetraacetic acid (DOTA), for radiolabeling.

A biophysical screening was conducted to identify probe combinations of shorter length with retained high affinity, high-specificity binding, efficient automated synthesis, and enhanced solubility of the secondary probe in aqueous buffer. The selected probes were radiolabeled with ^177^Lu and underwent in vitro assessments for specificity, affinity, and cellular processing. Combinations that excelled in the in vitro evaluation were subsequently examined in biodistribution studies in mice bearing HER2-expressing SKOV-3 xenograft and compared with the most effective pretargeting pair from our earlier study, Z-HP15:[^177^Lu]Lu-HP16.^29^

## 1 Results and Discussion

Our previously published preclinical therapy studies are based on PNA probes having 15 complementary nucleobases.^26,27^ Upon hybridization, these probes form an exceptionally stable duplex with a melting temperature (*T*_m_) in solution of 86–88 °C. In a Biacore experiment, less than 5% of the hybridized secondary PNA detached during a 17 h long dissociation phase.^20^ It is likely that shorter probe pairs than the original 15-mers provide enough binding affinity and specificity to achieve an efficient pretargeted radionuclide therapy in vivo. A redesigned second-generation 15-mer primary probe, Z_HER2_-HP15, together with a 9-mer complementary ^177^Lu-labeled secondary probe (HP16) was shown to provide more efficient pretargeting in vivo, with a significantly lower kidney uptake, than both the first-generation of 15-mer PNA probes and Z_HER2_-HP15 in combination with [^177^Lu]Lu-HP18 (15-mer) or [^177^Lu]Lu-HP17 (12-mer). In surface plasmon resonance (SPR), the interaction between Z_HER2_-HP15 and HP16 was characterized by a high (K_D_ = 280 pM) binding affinity with a very slow dissociation rate (*k*_d_ = 1.2 × 10^–5^ s^–1^), and in solution, a melting temperature of 73 °C was measured for the HP15:HP16 duplex.^29^

The primary goal of this investigation was to find shorter PNA-based pretargeting probes with a favorable tumor-to-kidney uptake ratio in vivo. Additionally, we aimed to establish an efficient and robust protocol for the automated microwave-assisted synthesis of PNA-based hybridization probes, replacing the labor-intensive manual SPS method previously employed for the majority of the probes in our previous studies.^20,29^

### 1.1 Design and Automated Microwave-Assisted Synthesis of Shorter PNA-Based Hybridization Probes

Here, we introduce three new shorter PNA-based pretargeting primary probes, HP12 (12-mer), HP9 (9-mer), and HP6 (6-mer), along with three new secondary probes, HP20 (8-mer), HP21 (7-mer), and HP19 (6-mer) (refer to Figure 2 and Table 1). The probes were designed by shortening the oligonucleotide sequence in our previously published second-generation of PNA-based probes. Compared to dsDNA, shorter PNA:PNA duplexes are exceptionally stable. For instance, four distinct 8-mer PNA:PNA duplexes displayed notably high *T*_m_s in the 52–55 °C range, while dsDNA with the same oligonucleotide sequences had about 40 °C lower predicted *T*_m_s.^36^ In addition, PNAs are superior in the recognition of single-base mutations. For example, the introduction of a single mismatch in a 10-mer PNA:PNA duplex caused the thermal melting temperature to decrease from 70 °C to a *T*_m_ in the range of 52–54 °C, depending on the type of mismatch introduced.^37^

**Table 1:** Sequences and Molecular Weights of the PNA-Based Probes^a^

name	reference	sequence	theoretical mass (Da)	experimental mass (Da)
HP15	(29)	GSS-**cc**t**g**g**t**gttgatgat-EK(DOTA)-AEEA-E-NH_2_	5274	5272
HP12	new	GGGSS-cctggtgttgat-EK(DOTA)-AEEA-E-NH_2_	4556	4558
HP9	new	GGGSS-**cctg**gtgtt-EK(DOTA)-AEEA-E-NH_2_	3722	3726
HP6	new	GGGSS-**cc**tggt-EK(DOTA)-AEEA-E-NH_2_	2899	2903
HP18	(29)	DOTA-AEEA-SS-**atc**atcaacaccagg-EEY-NH_2_	5168	5163
HP17	(29)	DOTA-AEEA-SS-atcaacaccagg-EEY-NH_2_	4375	4381
HP16	(29)	DOTA-AEEA-SS-aacaccagg-EEY-NH_2_	3583	3586
HP20	new	DOTA-AEEA-SS-acaccagg-EEY-NH_2_	3307	3310
HP21	new	DOTA-AEEA-SS-caccagg-EEY-NH_2_	3032	3033
HP19	new	DOTA-AEEA-SS-accagg-EEY-NH_2_	2780	2784

a In the table, amino acids are represented in uppercase letters, while PNA monomers are denoted in lowercase letters. Residues that necessitated double coupling are highlighted in bold font, and those that required triple coupling before obtaining a negative ninhydrin test are indicated in bold font with underlining. The table also includes information about the theoretical mass and the experimental mass, as determined by MALDI-TOF (refer to Figures S2–S11).

A lower limit of six complementary PNA bases was set with the HP6:HP19 pair. This decision was guided by the (limited) melting temperature data available from previous published studies on short PNA:PNA duplexes. Data on 6-mer PNA:PNA duplexes have consistently shown *T*_m_s above body temperature, typically falling within the range of 40–47 °C depending on sequence,^38^ while 5-mer duplexes have exhibited *T*_m_s at or below 30 °C.^39^

The application of microwave heating in the automated synthesis of PNA molecules has previously demonstrated several advantages, including improved yields and purer synthesis products compared to standard protocols. This method results in fewer truncated sequences, which can otherwise pose challenges during downstream purification processes. Notably, a microwave-heated protocol, employing diispropylcarbodiimide (DIC) and OxymaPure as coupling agents, has also shown a significant advantage in terms of speed, as reported in previous research.^35^ A protocol for the synthesis of HP15 had previously been developed using a Biotage Initiator + Alstra microwave peptide synthesizer^29^ and served as a starting point for an optimized synthesis protocol. In this study, we successfully synthesized new secondary probes (HP20, HP21, and HP19) and primary probes (HP12, HP9, and HP6) in addition to those examined in^29^ (refer to Table 1 for details).

Amino acids and the linker molecule {2-[2-(Fmoc-amino)ethoxy]ethoxy}acetic acid (AEEA) were coupled with 6 equiv of amino acid monomer or AEEA, along with 6 equiv of Oxyma and DIC in DMF, using a standard 10 min coupling at 75 °C. However, when coupling PNA monomers, modifications were needed. PNA stock solutions were prepared at 0.2 M in DMF, except for residue C, which required DMF:NMP (1:1) for good dissolution. This led to a final concentration of 0.07 M during coupling, with 4 equiv of PNA monomer used for couplings due to volume constraints.

We chose to maintain a reaction temperature at 75 °C^29^ to avoid potential side reactions, even though a higher temperature could speed up amide bond formation by lowering the activation energy. Additionally, we adhered to the DIC/OxymaPure coupling reagent combination, recognized for its efficiency in microwave-assisted amide bond formation.^35,40,41^ OxymaPure poses less risk of explosion than other coupling additives such as 1-hydroxybenzotriazole (HOBt),^40^ and the DIC/OxymaPure combination has proven to be especially suitable for the synthesis of aggregation-prone peptides and PNA molecules.^35,42^

For secondary probes, single couplings were typically sufficient for sequences with up to 12 PNA residues. However, for the 15-mer HP18, the last three PNA blocks (A, T, and C) needed double couplings. The radiometal chelator was coupled to the N-terminus with 8 equiv of DOTA-OtBu in NMP, 8 equiv of benzotriazol-1-yloxytripyrrolidinophosphonium hexafluorophosphate (PyBOP) in DMF, and 8 equiv of diisopropylethylamine (DIEA) at room temperature for 1.5 h. For sequences of the secondary probes and number of couplings at each position, refer to Table 1.

The synthesis of the primary probes involved sequence-specific modifications. Table 1 offers a summary of the number of couplings required for each residue in the primary probes. Furthermore, DOTA was linked to the side chain of the lysine residue after removing the 4-methyltrityl (Mtt)-protecting group using the same protocol as for the secondary probes. For a representative RP-HPLC chromatogram obtained during the purification of the crude synthesis product of the longest 15-mer primary probe, HP15, please refer to Figure S1.

### 1.2 Production of Affibody–PNA Chimeras

HP15, HP12, HP9, and HP6 were successfully conjugated to the affibody Z_HER2:342_-SR-H_6_ through the utilization of Sortase A 7+,^43^ a Ca^2+^-independent Sortase A variant. The purity of both purified Z_HER2:342_-SR-H_6_ and Sortase A 7+ is showcased in Figures S12 and S13, respectively, and a representative SDS-PAGE gel of the Sortase A-mediated coupling, and subsequent IMAC purification, of Z-HP12 can be found in Figure S14. The final purity of the secondary probes and the affibody-PNA conjugates used for in vitro cell studies and for in vivo experiments was determined through analytical RP-HPLC and mass spectrometry (MALDI-TOF or ESI-TOF) (refer to Figures S6–S11 for PNA probe purity and Figures S15–S18 for purity of affibody-PNA conjugates).

### 1.3 Solubility of PNA-Based Secondary Probes

PNA exhibits relatively low solubility in water and has a propensity to form self-aggregates. This behavior is influenced by factors such as oligomer chain length and sequence. PNA aggregates have been shown to interact nonspecifically with proteins and oligonucleotides,^44,45^ which can impair their usefulness for in vivo applications such as gene editing.^46^

The solubility of our secondary probes of different lengths, including HP18 (15-mer), HP17 (12-mer), HP16 (9-mer), and HP20 (8-mer), was assessed in a buffer used for lutetium-177 labeling (0.2 M NH_4_Ac; pH 5.5) at 25 °C (see Figure S19). Among these probes, the longest variant, HP18 (15-mer), exhibited the lowest solubility, measuring at just 3 μM. However, as we moved to shorter PNA oligomers, solubility saw a substantial, nonlinear increase. HP17 (12-mer), HP16 (9-mer), and HP20 (8-mer) exhibited approximately 2- (6 μM), 10- (26 μM), and a 100-fold increase (370 μM) in solubility, respectively, compared to the 15-mer HP18. It is worth noting that the ^177^Lu-labeling of the secondary probes is conducted at 95 °C,^47^ where the solubility of PNA is significantly improved compared to at 25 °C.^31^ For example, at 25 °C, the maximum soluble concentration of HP17 (12-mer) was around 6 μM. However, after 5 min at 95 °C, this solubility exceeded 82 μM (as shown in Figure S19C).

It is important to note that good solubility in an aqueous solution is a substantial precondition for a successful clinical translation. First, it enables a relatively easy formulation of a lyophilized kit for labeling, facilitating an implementation in clinical radiopharmacy. Second, the success of pretargeting depends on the correct ratio between masses of injected primary and secondary probes.^26^ Good solubility prevents underdosing of the secondary probe.

### 1.4 Biophysical Characterization of PNA-Based Pretargeting Probes Using SPR, UV-Melting Analysis and Circular Dichroism (CD)

The CD spectra of Z-HP12 and Z-HP9 (Figure S20A,C) showed characteristic minima around 222 and 208 nm, indicating proteins with a high helical content, and spectra taken at 20 °C both before and after thermal denaturation at 95 °C suggested that the proteins can refold after thermal melting. Both proteins exhibited similar melting temperatures, with Z-HP12 at 65 °C and Z-HP9 at 66 °C (Figure S20B,D), in line with the melting temperature of 67 °C previously determined for the Z_HER2:342_ parental molecule.^8^ The equilibrium dissociation constants (*K*_D_s) for Z-HP12 and Z-HP9 binding to HER2-Fc were measured to be 180 and 280 pM, respectively, using SPR (refer to Figure S21 and Table S1 in the Supplementary data). These measured kinetic constants align excellently with those obtained for both the first-generation Z-HP1:HP2 (*K*_D_= 212 pM^20^) and the second-generation Z-HP15:HP18 (*K*_D_ = 276 pM^29^) of hybridized 15-mer pretargeting agents binding to the HER2 receptor. Additionally, they are consistent with dissociation equilibrium constants (90.2–283 pM) reported earlier for Z_HER2:342_ variants with short peptide extensions at the C-terminal.^48^ Therefore, neither the length nor sequence of the PNA-based primary probe, nor the hybridization to the secondary probe, has more than a marginal effect on the binding affinities to the HER2 receptor.

**Figure 3:**
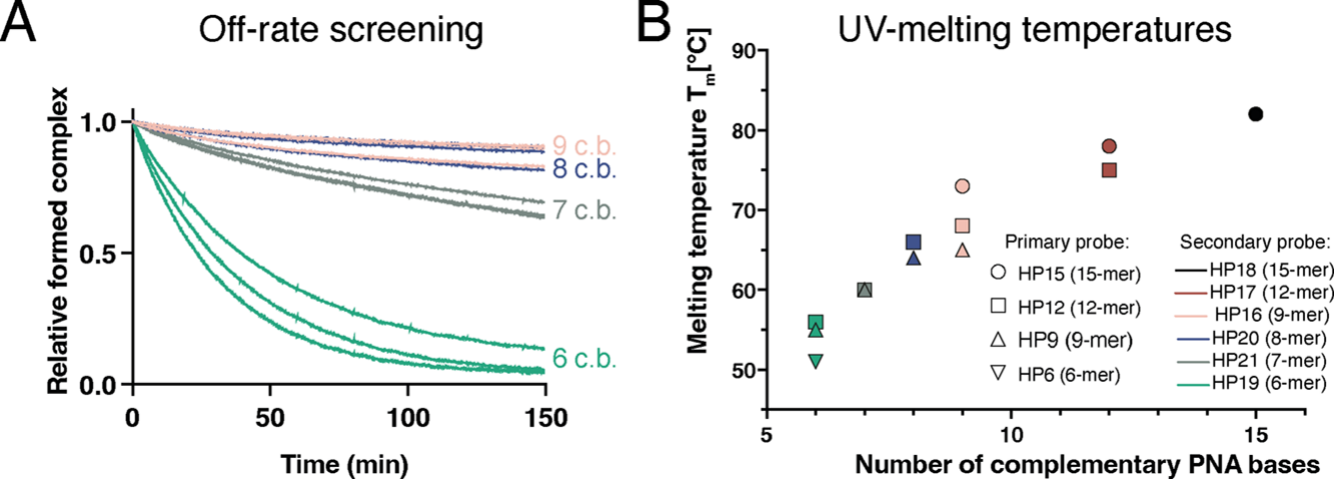
(A) Off-rate screening by SPR of different probe complexes. The color indicates the number of complementary bases (c.b.) in each formed complex. (B) Melting temperature in solution of unconjugated PNA-based probe pairs, ordered by the number of complementary bases in each duplex. Dissociation rates and melting temperatures for the different duplexes are given in Table 2.

The stability of new pretargeting probe combinations was assessed using SPR, and duplex melting temperatures were measured with UV–vis spectroscopy. In SPR, a high concentration of the secondary probe was injected over immobilized primary agents, and the dissociation of the hybridized complexes was monitored over 150 min (see Figures 3A, S22, and Table 2). Z-HP15 and Z-HP12 in conjunction with HP16 (9-mer) and HP20 (8-mer) exhibited very slow dissociation rates (*k*_d_ = 1.2–1.6 × 10^–5^ s^–1^), close to the detection limit of the T200 SPR instrument (*k*_d_ ≤ 10^–5^ s^–1^). A modest ∼2-fold increase in *k*_d_ was observed for corresponding complexes with the shorter Z-HP9 primary probe (*k*_d_ = 2.5–2.6 × 10^–5^ s^–1^). Complexes formed between Z-HP15, Z-HP12, and Z-HP9 and the shorter 7-mer and 6-mer secondary probes showed ∼4-fold (*k*_d_ = 4.5–9.2 × 10^–5^ s^–1^) and ∼20-fold (*k*_d_ = 2.8–6.2 × 10^–4^ s^–1^) faster off-rates, respectively.

**Table 2:** Dissociation Rate Constants (*k*_d_s) and Melting Temperatures (*T*_m_s) of the PNA Probe Duplexes^a^

secondary probe	complementary bases	primary probe	*k*_d_ (s^–1^)	*T*_m_ (°C)
HP18	15	HP15	<10^–5^^b^	82
HP17	12	HP15	<10^–5^^b^	78
		HP12	n.d.	75
HP16	**9**	**HP15**	1.2 × 10^–5^	**73**
		**HP12**	**1.3 × 10^–5^**	**68**
		**HP9**	**2.5 × 10^–5^**	**65**
HP20	**8**	HP15	1.4 × 10^–5^	n.d.
		**HP12**	**1.6 × 10^–5^**	**66**
		**HP9**	**2.6 × 10^–5^**	**64**
HP21	7	HP15	5.5 × 10^–5^	n.d.
		HP12	4.5 × 10^–5^	60
		HP9	9.2 × 10^–5^	60
HP19	6	HP15	5.0 × 10^–4^	n.d.
		HP12	6.2 × 10^–4^	56
		HP9	2.8 × 10^–4^	55
		HP6	n.d.	51

a The *k*_d_s were obtained through SPR by fitting the dissociation phase of the sensorgrams in Figure 5 to a 1:1 binding model using the Biacore T200 instrument software. Melting temperatures were assessed by monitoring the absorbance at 260 nm as a function of temperature. Combinations highlighted in bold were chosen for subsequent in vitro testing.

b Result from^29^, n.d. = not determined.

The melting temperatures of secondary probes after duplex formation with primary probes were also measured by monitoring the absorbance at 260 nm as a function of temperature (see Figure S23). All probe pairs had melting temperatures well above 37 °C (body temperature) and ranged from 51 °C for the HP6:HP19 pair with 6 complementary bases to 82 °C for the previously studied HP15:HP18 pair with 15 complementary bases (see Figure 3B and Table 2). It is noteworthy that all the studied duplexes exhibited the CD signal characteristic of left-handed P helices (refer to Figure S24). This secondary structure is typically adopted by antiparallel and L-amino acid-modified PNA–PNA duplexes at neutral pH.^49^

Both SPR and thermal UV-melt data indicate that the stability of complexes is linked to the length of their complementary sequences. Additionally, an increase in melting temperature and a decrease in *k*_d_ were observed for PNA–PNA duplexes with unpaired overhanging nucleobases in the primary probe. For instance, within the group of PNA:PNA duplexes with 9 complementary bases, the duplex with a 6-nucleotide overhang in the primary probe, HP15:HP16, displayed the highest melting temperature (73 °C). This temperature decreased to 68 °C for a 3-nucleotide overhang in HP12:HP16 and further to 65 °C for the length-matched 9-base pair HP9:HP16 duplex. This phenomenon, referred to as the ‘overhang effect’, has been previously observed for PNAs binding to longer DNA^50^ or PNA^39^ molecules, and emphasize the importance of individually evaluating each PNA–PNA probe pair, even if they share the same hybridizing sequence.

A comprehensive multicycle kinetic (MCK) SPR analysis was conducted at both 25 and 37 °C on the Z-HP9:HP16 and Z-HP9:HP20 pairs in comparison to the previously investigated Z-HP15:HP16 pair (see Figure S25 and Table 3). The observed ∼2-fold increase in the dissociation rate constant (*k*_d_) for complexes formed with Z-HP9, compared to the corresponding Z-HP12 and Z-HP15 pairs, prompted further investigation to understand its impact on binding affinity and stability. The Z-HP15:HP16 pair showed a *K*_D_ of 320 pM at 25 °C, consistent with our previous findings (280 pM^29^). It displayed the slowest association rate constant (*k*_a_) among the three studied pairs at 1.0 × 10^5^ M^–1^ s^–1^, as well as the slowest dissociation rate constant (*k*_d_) at 3.4 × 10^–5^ s^–1^. The off-rate increased by approximately 2-fold to 6.6 × 10^–5^ s^–1^ for the Z-HP9:HP20 complex, resulting in a *K*_D_ value of 550 pM for the binding of Z-HP9 to the 8-mer at 25 °C.

**Table 3:** Kinetic Parameters of PNA Probe Hybridisation Determined by SPR

secondary probe	complementary bases	primary probe	*k*_a_ (M^–1^ s^–1^)	*k*_d_ (s^–1^)	*K*_D_ (pM)	χ^2^	temperature (°C)
HP16	9	HP15	1.0 × 10^5^	3.4 × 10^–5^	320	0.4	25
			1.8 × 10^5^	8.1 × 10^–5^	440	0.5	37
		**HP9**	1.8 × 10^5^	4.8 × 10^–5^	270	2.4	25
			2.9 × 10^5^	1.1 × 10^–4^	390	2.4	37
HP20	8	**HP9**	1.2 × 10^5^	6.6 × 10^–5^	550	0.5	25
			2.2 × 10^5^	1.7 × 10^–4^	790	1.0	37

The length-matched 9-mer Z-HP9:HP16 pair exhibited slightly faster on- (1.8 × 10^5^ M^–1^ s^–1^) and off- (4.8 × 10^–5^ s^–1^) rates but maintained a similar *K*_D_ (270 pM) compared to the Z-HP15:HP16 pair. While the dissociation constants measured by SPR remained similar, thermal melt data in solution indicate that the 6-base overhang in HP15:HP16 has a stabilizing effect on the duplex when compared to the length-matched HP9:HP16 pair. This results in an 8 °C difference in melting temperature.

Interestingly, elevating the temperature to 37 °C had a similar effect on the binding affinity for all three probe pairs. Off-rates increased approximately 2.5-fold, but were compensated by a 1.7-fold increase in on-rates, resulting in a small (∼1.4-fold) overall decrease in *K*_D_ at 37 °C compared to 25 °C.

Even in the case of the weakest binding pair, Z-HP9:HP20, as determined through MCK SPR analysis, a subnanomolar *K*_D_ of 550 pM at 25 °C and 790 pM at 37 °C was maintained. In comparison, the previously studied pair, Z-HP15:HP16, exhibited a slightly lower *K*_D_ of 320 pM at 25 °C and 440 pM at 37 °C. Notably, an extremely high binding affinity of 11.4 ± 0.4 pM has previously been determined for the Z-HP15:HP16 pair. This affinity was measured using LigandTracer analysis on SKOV-3 cells with a ^177^Lu-labeled HP16 secondary probe, binding to cells that had been pretreated with Z-HP15.^29^

Taken together, we chose to proceed with cell-based studies using the Z-HP12 (12-mer) and Z-HP9 (9-mer) primary probes in combination with the HP16 (9-mer) and HP20 (8-mer) secondary probes. The duplexes formed between the 9-mer and 8-mer secondary probes and HP12 and HP9 are all thermally stable (*T*_m_ = 64–68 °C) and exhibit similar slow off-rates as observed with the Z-HP15:HP16 pair, which had previously shown excellent pretargeting in vivo.^29^

### 1.5 Radiolabeling of Probes with ^177^Lu and In Vitro Stability

Table 4 summarizes the radiochemical yield (RCY), purity, and stability test results for all ^177^Lu-labeled probes in this study. The radiolabeling of primary agents, Z-HP9 and Z-HP12, yielded radiochemical purities of 29.1 ± 6.6 and 17.1 ± 2.5%, respectively. Such radiolabeling yield necessitated purification to achieve the requisite radiochemical purity of over 95%. Purification was conducted using a NAP-5 column, eluted with 1% BSA in PBS, resulting in radiochemical purities of 99 ± 1.7% for [^177^Lu]Lu-Z-HP9 and 98.5 ± 2.2% for [^177^Lu]Lu-Z-HP12, respectively. The relatively low yield of the primary probes was most likely caused by the presence of free chelator. In this study, the labeling of primary probes Z-HP9 and Z-HP12 was used to evaluate quantitatively in vitro specificity and affinity of their binding to HER2-expressing cells and their cellular processing and retention. Thus, it was essential that the label was stable, and the compound was pure. Therefore, we did not optimize the labeling of the primary probes. The RCY for the secondary probes exceeded 97%, and consequently, further purification was deemed unnecessary for in vitro and in vivo studies. Importantly, all probes labeled with ^177^Lu remained stable in the presence of an excess amount of EDTA and in PBS as control after incubation at 37 °C for 1 h.

**Table 4:** ^177^Lu-Labeling of PNA-Based Probes and In Vitro Stability

probes	radiochemical yield, %	isolated yield, %	radiochemical purity, %	stability in PBS, 37 °C, 1 h	stability (×500 EDTA), 37 °C, 1 h
[^177^Lu]Lu-Z-HP9	29.1 ± 6.6	19.1 ± 5.2	99 ± 1.7	97 ± 2	96.3 ± 2.1
[^177^Lu]Lu-Z-HP12	17.1 ± 2.5	11.3 ± 3.2	98.5 ± 2.2	100 ± 0	100 ± 0.0
[^177^Lu]Lu-HP16	97.2 ± 1.1			100 ± 0^a^	100 ± 0^a^
[^177^Lu]Lu-HP20	99.4 ± 0.5			100 ± 0	99.5 ± 0.7

a Result from ref (29).

The accuracy of the radiolabeling of secondary probes was confirmed through cross-validation using radio-HPLC analysis. The radio-HPLC radiochromatogram (see Figure S26) demonstrated that both [^177^Lu]Lu-HP16 and [^177^Lu]Lu-HP20 exhibited retention times of approximately 6 min. Notably, only a single major peak was observed, which corresponded to the radiolabeled compound. The first minor peak was identified as free Lu-177.

### 1.6 In Vitro Cell Studies

The results of the binding affinity of all radiolabeled probes to SKOV-3 cells are illustrated in Figure S27 and summarized in Table 5. Based on LigandTracer measurements and InteractionMap calculations, the optimal fit for the binding of the radioconjugates to the SKOV-3 cell line was achieved using a 1:2 model, indicating the presence of two types of interactions with the HER2 target.

**Table 5:** Equilibrium Dissociation Constants (*K*_D_) of ^177^Lu-Labeled Probes on HER2-Expressing SKOV-3 Cells Determined Using an InteractionMap Analysis

probe	*K*_D1_ (pM)	weight (%)	*K*_D2_ (nM)	weight (%)
[^177^Lu]Lu-Z-HP9	83.4 ± 11.1	92.05 ± 0.84	6.87 ± 1.56	7.95 ± 0.84
[^177^Lu]Lu-Z-HP12	49.9 ± 4.8	95.11 ± 0.72	4.83 ± 0.64	4.89 ± 0.72
Z-HP9:[^177^Lu]Lu-HP16	10.8 ± 3.3	78.16 ± 5.76	0.76 ± 0.21	21.84 ± 5.76
Z-HP12:[^177^Lu]Lu-HP16	32.6 ± 26.2	76.77 ± 3.46	2.44 ± 0.23	23.23 ± 3.46
Z-HP9:[^177^Lu]Lu-HP20	10.9 ± 2.3	81.45 ± 1.97	6.15 ± 2.23	18.55 ± 1.97
Z-HP12:[^177^Lu]Lu-HP20	N.B^a^	N.B	N.B	N.B
Z-HP15:[^177^Lu]Lu-HP20	28.0 ± 6.5	76.89 ± 5.52	2.79 ± 2.11	23.11 ± 5.52

a N.B = no binding.

Concerning the affinity of individual primary agents, [^177^Lu]Lu-Z-HP12 exhibited a higher affinity (*K*_D1_ = 49.9 ± 4.8 pM, weight %=95) than [^177^Lu]Lu-Z-HP9 (*K*_D1_ = 83.4 ± 11.1 pM, weight %=92). Surprisingly, despite Z-HP12’s higher affinity (49.9 ± 4.8 pM), [^177^Lu]Lu-HP20 exhibited no binding to cells pretrated with Z-HP12. This was in stark contrast to the high-affinity binding observed when the same primary agent was paired with [^177^Lu]Lu-HP16 (*K*_D1_ = 32.6 ± 26.2 pM, weight % = 77). The exact reasons for this inconsistency, possibly stemming from a failed hybridization between [^177^Lu]Lu-HP20 and Z-HP12, were not further elucidated. Consequently, the Z-HP12 primary agent was excluded from subsequent in vivo assays.

Regarding the affinity of ^177^Lu-labeled secondary probes to the cell pretreated with primary agents, it was observed that both ^177^Lu-labeled secondary probes (HP16 and HP20) in combination with Z-HP9 as the primary agent exhibited similarly high-affinity values (*K*_D1_ = 10.8 ± 3.3 pM for Z-HP9:[^177^Lu]Lu-HP16 and *K*_D1_ = 10.9 ± 2.3 pM for Z-HP9:[^177^Lu]Lu-HP20). Thus, the overall affinity to Z-HP9-pretreated cells, whether using a 9-mer ([^177^Lu]Lu-HP16) or an 8-mer ([^177^Lu]Lu-HP20) probe, was comparable. Importantly, these affinity values were similar to the previously studied Z-HP15:[^177^Lu]Lu-HP16 pair (*K*_D_ = 11.4 ± 0.4 pM).^29^ Consequently, transitioning from a longer (Z-HP15) to a shorter (Z-HP9) primary probe had no significant impact on the binding affinity of the [^177^Lu]Lu-HP16 probe in the cell-based assay. This aligns with a previous study, which found no discernible effect on the apparent binding affinity for three different lengths of ^177^Lu-labeled secondary probes (15-mer, 12-mer, and 9-mer) binding to HER2-positive cells pretreated with Z-HP15.^29^

**Figure 4:**
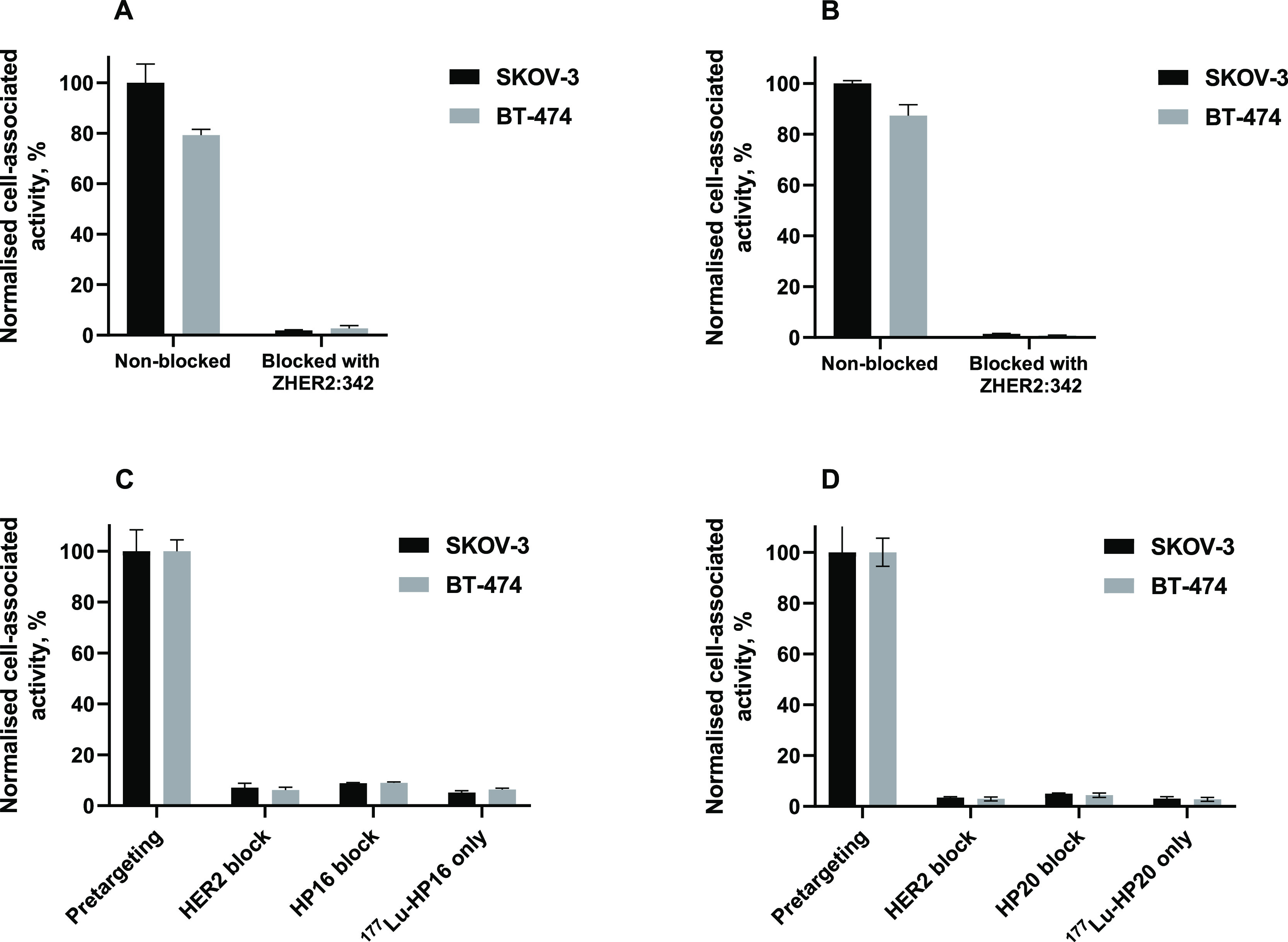
In vitro binding specificity of primary agents, (A) [^177^Lu]Lu-Z-HP9 and (B) [^177^Lu]Lu-Z-HP12 on SKOV-3 and BT-474 cell lines. Binding specificity of secondary agents (C) [^177^Lu]Lu-HP16 and (D) [^177^Lu]Lu-HP20, the cells in the first group were preincubated with Z-HP9 before the addition of a radiolabeled secondary agent. In the second and third groups, cells were incubated with a large amount of unlabeled anti-HER2 Z_HER2:342_ affibody molecule and unlabeled secondary agent before the addition of radiolabeled secondary agent, respectively. A radiolabeled secondary agent was added directly to cells in the fourth group without adding a primary agent. The data are presented as an average value from three samples ±SD.

The in vitro binding specificity of the new PNA-based probes was assessed through a saturation experiment, with the results displayed in Figure 4. Notably, the binding of the primary agents, [^177^Lu]Lu-Z-HP9 and [^177^Lu]Lu-Z-HP12, to SKOV-3 and BT-474 cells was significantly reduced (*p* < 0.05) after HER2 receptors were saturated with an excess of unlabeled anti-HER2 affibody molecules, as depicted in Figure 4A,B. These results provide clear evidence of the HER2-specific nature of the binding of the primary agents.

Figure 4C,D further elucidates the specificity of PNA-mediated pretargeting on HER2-expressing cell lines when cells were pretreated with a primary agent. The binding of both [^177^Lu]Lu-HP16 and [^177^Lu]Lu-HP20 to Z-HP9 pretreated cells was 11-fold higher than that of other treated groups. Significantly, the uptake of both [^177^Lu]Lu-HP16 and [^177^Lu]Lu-HP20 was notably reduced when the binding of Z-HP9 was blocked by an excess of anti-HER2 affibody molecule Z_HER2:342_ and nonlabeled secondary probes. This clearly demonstrates that the binding of radiolabeled secondary probes is mediated by HER2 and PNA, respectively. Additionally, the binding of all secondary agents to cells without preincubation with primary agents was significantly lower (*p* < 0.05) than in the pretargeting scenario. These findings collectively affirm the success of the in vitro pretargeting approach.

**Figure 5:**
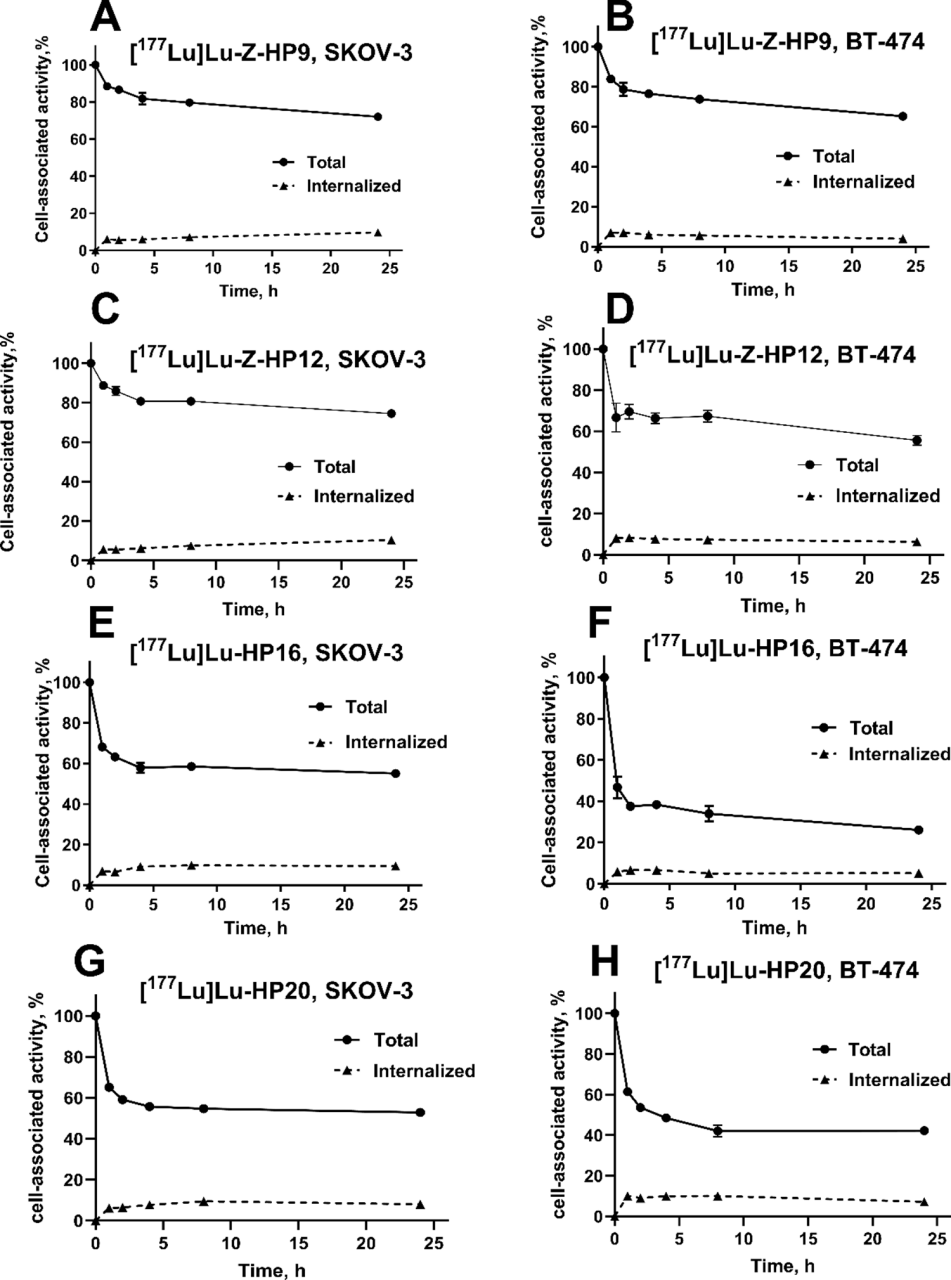
Cellular processing and retention of [^177^Lu]Lu-Z-HP9 (A,B), [^177^Lu]Lu-Z-HP12 (C,D), [^177^Lu]Lu-HP16 (E,F), and [^177^Lu]Lu-HP20 (G, H) on SKOV-3 (A,C,E,G) and BT-474 (B,D,F,H) cells after interrupted incubation with labeled compounds. For the ^177^Lu-labeled secondary probes, the cells were pretreated with 1 nM of nonlabeled Z-HP9. The data are presented as an average value from three samples ±SD.

Figure 5 displays the cellular processing and retention data for the ^177^Lu-labeled probes on SKOV-3 and BT-474 cells. After a 24 h incubation period, the total cell-associated bound activity was recorded as 72.0 ± 1.7 and 65.2 ± 1.8% for [^177^Lu]Lu-Z-HP9, and 74.5 ± 0.2 and 55.6 ± 2.4% for [^177^Lu]Lu-Z-HP12 on SKOV-3 and BT-474 cells, respectively. The internalized fractions were quantified as 9.7 ± 1.1 and 10.4 ± 0.6% for [^177^Lu]Lu-Z-HP9 and [^177^Lu]Lu-Z-HP12 after 24 h of incubation. The pattern was similar for both HER2-expressing cell lines, with slow internalization, which is typical for affibody molecules.

The pattern of internalization of the labeled secondary probes by cancer cells, which were pretreated with primary probes, was similar to the pattern for primary agents. After a 24 h incubation period, the total cell-associated bound activity was 55.0 ± 1.0 and 26.1 ± 1.2% for [^177^Lu]Lu-HP16, and 52.8 ± 0.4 and 42.2 ± 2.0% for [^177^Lu]Lu-HP20 on SKOV-3 and BT-474 cells, respectively. However, it is worth noting that the retention of radioactivity over time was more pronounced for ^177^Lu-labeled primary agents, while the ^177^Lu-labeled secondary probes exhibited a relatively faster release of bound radioactivity from the cells as time progressed.

### 1.7 In Vivo Studies

The comparison of biodistribution between [^177^Lu]Lu-HP16 and [^177^Lu]Lu-HP20, without prior injection of the nonlabeled Z-HP9 primary agent at the 4 h postinjection time point, is presented in Table S2. The organ uptakes between both groups were somewhat similar. However, [^177^Lu]Lu-HP20 had significantly (*p* < 0.05) lower muscle uptake than for [^177^Lu]Lu-HP16.

**Figure 6:**
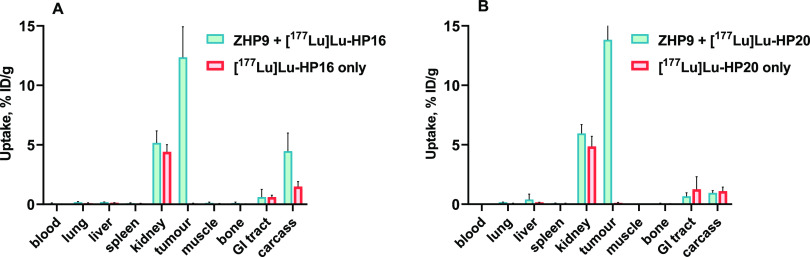
In vivo specificity of (A) [^177^Lu]Lu-HP16 and (B) [^177^Lu]Lu-HP20 in BALB/C nu/nu mice bearing SKOV-3 xenografts at 4 h p.i. with and without preinjection of primary agent, Z-HP9. Data are presented as an average of %ID/g ± SD, *n* = 4.

Figure 6 illustrates the results of in vivo specificity testing for [^177^Lu]Lu-HP16 and [^177^Lu]Lu-HP20 with preinjection of the primary agent Z-HP9 (45 μg, 4 nmol) at 4 h postinjection. When mice were preinjected with the Z-HP9 primary agent, the tumor uptake of both [^177^Lu]Lu-HP16 and [^177^Lu]Lu-HP20 secondary probes demonstrated a significant (*p* < 0.05) increase compared to the group without preinjection, underscoring the HER2-specific and PNA-mediated nature of the interaction. It is noteworthy that in the group of mice preinjected with the primary agent, there was higher uptake in other organs compared to the group without preinjection of the primary agent. One plausible explanation for this elevated uptake after pretreatment with the primary affibody-PNA conjugate could be hybridization with incompletely cleared primary agent or primary agent that has dissociated from the tumor and re-entered the bloodstream. However, it is important to note that the level of uptake still remained relatively low, even after preinjection with the primary agent.

The results of the comparative biodistribution of [^177^Lu]Lu-HP16 (previously studied in ref (29)) and [^177^Lu]Lu-HP20 (the new secondary agent) with preinjection of the new primary agent Z-HP9 and Z-HP15 (used as a comparator), in mice bearing HER2-expressing SKOV-3 xenografts at 4 and 144 h postinjection, are presented in Table 6. Biodistribution measurements of all radiolabeled PNA-based probes indicated rapid clearance from the blood as well as from other organs and tissues, which is a crucial requirement for the success of in vivo pretargeting strategies. Notably, the only tissues exhibiting substantial uptake were the tumor and kidney.

**Table 6:** Biodistribution of ^177^Lu-Labeled Secondary Agents HP16 and HP20 Preincubated with Primary Agents Z-HP9 and Z-HP15 in BALB/C nu/nu Mice Bearing SKOV-3 Xenografts at 4 and 144 h Postinjection^a^

site	uptake, %ID/g					
	4 h			144 h		
	Z-HP9: [^177^Lu]Lu-HP16	Z-HP9: [^177^Lu]Lu-HP20	Z-HP15: [^177^Lu]Lu-HP16	Z-HP9: [^177^Lu]Lu-HP16	Z-HP9: [^177^Lu]Lu-HP20	Z-HP15: [^177^Lu]Lu-HP16
blood	0.08 ± 0.04	0.04 ± 0.004	0.07 ± 0.02	0.004 ± 0.001	0.002 ± 0.002	0.001 ± 0.001
lung	0.18 ± 0.05	0.16 ± 0.02	0.14 ± 0.02	0.015 ± 0.007	0.013 ± 0.015	0.011 ± 0.013
liver	0.19 ± 0.01^c^	0.17 ± 0.01	0.13 ± 0.04^c^	0.045 ± 0.015^b^	0.074 ± 0.007^b^^,^^d^	0.046 ± 0.006^d^
spleen	0.10 ± 0.03	0.08 ± 0.02	0.08 ± 0.02	0.030 ± 0.014	0.031 ± 0.009	0.033 ± 0.01
kidney	5.16 ± 1.02	5.95 ± 0.75	5.36 ± 1.23	0.475 ± 0.118	0.478 ± 0.094	0.458 ± 0.064
tumor	12.36 ± 2.57^c^	13.82 ± 1.21	16.55 ± 1.76^c^	1.912 ± 0.381	1.795 ± 0.073	1.747 ± 0.244
muscle	0.12 ± 0.07^b^^,^^c^	0.03 ± 0.02^b^	0.040 ± 0.002^c^	0.011 ± 0.007	0.014 ± 0.002	0.007 ± 0.008
bone	0.11 ± 0.08	0.06 ± 0.02	0.09 ± 0.01	0.029 ± 0.013	0.036 ± 0.010	0.047 ± 0.048
GI^e^	0.61 ± 0.65	0.67 ± 0.29	1.01 ± 0.78	0.062 ± 0.028	0.047 ± 0.023	0.045 ± 0.008
Carcass^e^	4.47 ± 1.52	0.95 ± 0.19	3.23 ± 2.2	0.101 ± 0.034	0.084 ± 0.012	0.147 ± 0.016

a Data are presented as an average of %ID/g ± SD, *n* = 4.

b Significant difference (*p* < 0.05) between Z-HP9:[^177^Lu]Lu-HP16 and Z-HP9:[^177^Lu]Lu-HP20.

c Significant difference (*p* < 0.05) between Z-HP9:[^177^Lu]Lu-HP16 and Z-HP15:[^177^Lu]Lu-HP16.

d Significant difference (*p* < 0.05) between Z-HP9:[^177^Lu]Lu-HP20 and Z-HP15:[^177^Lu]Lu-HP16.

e The gastrointestinal (GI) and carcass data are expressed as %ID per whole sample. One-way ANOVA with Bonferroni’s multiple comparisons test was performed to find significant differences. ANOVA test (Bonferroni’s multiple comparisons test) was performed to test significant (*p* < 0.05) difference.

The biodistribution of [^177^Lu]Lu-HP16 and [^177^Lu]Lu-HP20 with preinjection of the Z-HP9 primary agent exhibited relatively similar patterns. At 4 h postinjection, the tumor uptake of [^177^Lu]Lu-HP16 preinjected with Z-HP15 (16.55 ± 1.76%ID/g) was significantly higher (*p* < 0.05) than the tumor uptake of [^177^Lu]Lu-HP16 preinjected with Z-HP9 (12.36 ± 2.57%ID/g). Hepatic uptake was significantly lower (*p* < 0.05) for [^177^Lu]Lu-HP16 pretreated with Z-HP15 (0.13 ± 0.04%ID/g) than for [^177^Lu]Lu-HP16 pretreated with Z-HP9 (0.19 ± 0.01%ID/g). There were no significant differences in activity uptake in any organs or tissues, including the tumor and liver, for [^177^Lu]Lu-HP20 pretreated with Z-HP9 (with uptake values of 13.82 ± 1.21%ID/g and 0.17 ± 0.01%ID/g in the tumor and liver, respectively) and the [^177^Lu]Lu-HP16:Z-HP15 pair. Muscle uptake of Z-HP9:[^177^Lu]Lu-HP16 (0.12 ± 0.07%ID/g) was significantly higher (*p* < 0.05) than for Z-HP9:[^177^Lu]Lu-HP20 (0.030 ± 0.015%ID/g) and Z-HP15:[^177^Lu]Lu-HP16 (0.040 ± 0.002%ID/g). Renal uptake was low for all groups, with no significant difference (5.16 ± 1.02, 5.95 ± 0.75, and 5.36 ± 1.23%ID/g for Z-HP9:[^177^Lu]Lu-HP16, Z-HP9:[^177^Lu]Lu-HP20, and Z-HP15: [^177^Lu]Lu-HP16, respectively).

At 144 h after injection, most of the uptake in organs and tissues, including tumor and kidney, was significantly (*p* < 0.05) reduced compared to 4 h p.i. Tumor and kidney remained the tissues with the highest activity uptake. Tumor-associated activity of [^177^Lu]Lu-HP16 and [^177^Lu]Lu-HP20 was reduced from 12.36 ± 2.57 and 13.82 ± 1.2%ID/g at 4 h after injection to 1.91 ± 0.831 and 1.79 ± 0.07%ID/g at 144 h after injection, respectively.

Hepatic uptake of [^177^Lu]Lu-HP16 pretreated with Z-HP9 (0.045 ± 0.015%ID/g) was significantly (*p* < 0.05) lower than for [^177^Lu]Lu-HP20 pretreated with the same Z-HP9 primary agent (0.074 ± 0.007%ID/g). There was significantly (*p* < 0.05) lower liver uptake for [^177^Lu]Lu-HP16 pretreated with Z-HP15 (0.046 ± 0.006%ID/g) than for [^177^Lu]Lu-HP20 pretreated with Z-HP9 (0.074 ± 0.007%ID/g).

**Figure 7:**
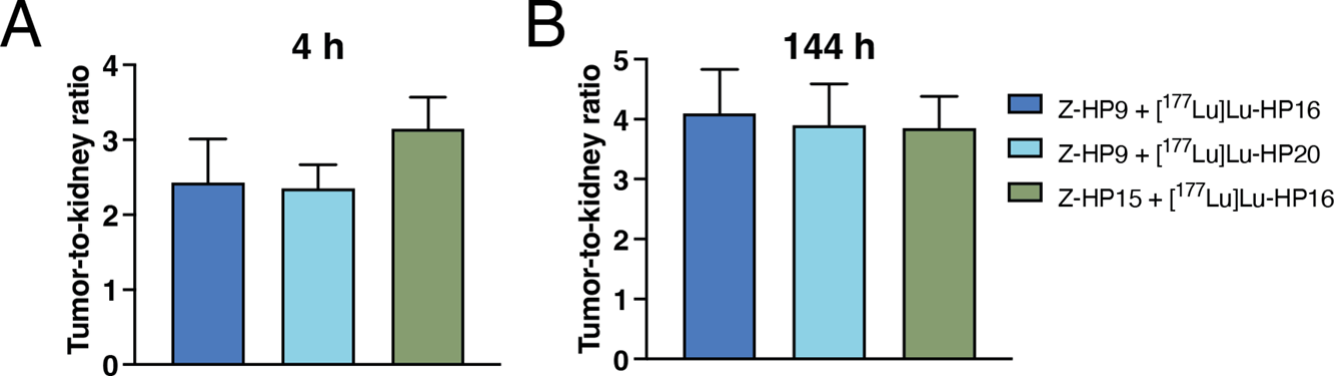
Comparison of tumor-to-kidney ratios of ^177^Lu-labeled secondary probes in BALB/C nu/nu mice bearing SKOV-3 xenografts at 4 (A) and 144 (B) h after injection. The uptake is expressed as % ID/g and presented as an average value from 4 mice ± SD.

The uptake of the tumor exceeded that of most normal tissues by several hundred-fold, with the kidneys being the dose-limiting organ critical for radionuclide therapy. Therefore, it is imperative that the absorbed dose in the tumor is significantly higher than in the kidneys to achieve successful pretargeting therapy. The tumor-to-kidney ratio is a key parameter, representing the ratio between the activity uptake in the targeted tumor tissue and the uptake in the dose-limiting kidneys. The calculated tumor-to-kidney ratios for all combinations are summarized in Figure 7 and Table 7.

**Table 7:** Comparison of Tumor-to-Kidney Ratios of ^177^Lu-Labeled Secondary Probes in BALB/C nu/nu Mice Bearing SKOV-3 Xenografts at 4 and 144 h Postinjection^a^

time	tumor-to-kidney ratio		
	Z-HP9:[^177^Lu]Lu-HP16	Z-HP9:[^177^Lu]Lu-HP20	Z-HP15:[^177^Lu]Lu-HP16
4 h	2.43 ± 0.58	2.35 ± 0.32	3.15 ± 0.42
144 h	4.1 ± 0.73	3.9 ± 0.69	3.85 ± 0.53

a Data are presented as an average of %ID/g ± SD, *n* = 4.

The tumor-to-kidney ratios were 2.43 ± 0.58, 2.35 ± 0.32, and 3.15 ± 0.42 for Z-HP9:[^177^Lu]Lu-HP16, Z-HP9:[^177^Lu]Lu-HP20, and the previously studied Z-HP15:[^177^Lu]Lu-HP16 pair, respectively. At 4 h postinjection, there were no significant differences in the tumor-to-kidney ratios among the three different pretargeting groups. Interestingly, at 144 h postinjection, the tumor-to-kidney ratios increased for all pretargeting groups and were 4.1 ± 0.73, 3.9 ± 0.69, and 3.85 ± 0.53 for Z-HP9:[^177^Lu]Lu-HP16, Z-HP9:[^177^Lu]Lu-HP20, and Z-HP15:[^177^Lu]Lu-HP16, respectively. No significant differences were observed among the three groups of mice. It should be noted that there is still a sufficient retention of activity in the tumor, although the tumor-associated activity of [^177^Lu]Lu-HP16 and [^177^Lu]Lu-HP20 has reduced dramatically at 144 h compared to 4 h p.i. Interestingly, there was also a quick washout of activity from the kidney over time. Such a washout from tumors could lead to insufficient delivery of high-dose activity in the tumors for radionuclide therapy. This could be due to secondary probes dissociating from the primary probes or the whole pretargeting system getting released from the tumor receptors. However, the use of Z-HP1:[^177^Lu]Lu-HP2 has demonstrated an extension of survival in mice bearing SKOV-3 xenograft, although the tumor-associated activity has reduced from 17 ± 3%ID/g at 4 h to 3.4 ± 0.6%ID/g at 144h.^26^ This suggests that radionuclide therapy using smaller primary and secondary probes might be feasible but with several additional cycles in order to have a full therapeutic effect.

The tumor-to-kidney ratio of the shorter [^177^Lu]Lu-HP20 secondary probe was as high as for [^177^Lu]Lu-HP16 pretreated with Z-HP15 and/or Z-HP9 at both time points. This shows that shortening the primary probe, either in combination with HP16 or shorter HP20 secondary probes, resulted in similar delivery of high-absorbed doses to tumor while avoiding high-absorbed dose to the critical organ, e.g., the kidney. Furthermore, compared to the first-generation of the pretargeting system, the tumor-to-kidney ratio for the new pretargeting pair (the shortest), Z-HP9:[^177^Lu]Lu-HP20 at 4 h (2.35 ± 0.32) in this study was ca. 1.2-fold higher than the tumor-to-kidney ratio for Z-HP1:[^177^Lu]Lu-HP2 (2.0 ± 0.4) at 4 h p.i. This ratio was similar for both pairs at 144 h p.i.^29^ Further reduction of the renal uptake might permit injection of higher activity (and enhance the treatment efficacy) without additional risk of renal toxicity. Competitive inhibition of the renal reabsorption of radiopeptides using positively charged amino acids or the plasma expander Gelofusine is used methods^54^ for kidney protection during peptide receptor radionuclide therapy. Animal studies have demonstrated that coinjection of l-lysine or Gelofusine reduced the renal uptake of the first-generation effector probe [^177^Lu]Lu-HP2.^47^ This might be an approach to test for the shorter effector probes as well.

Importantly, in terms of production and purification of the probes, it can be cost-efficient to produce smaller probes than the previous generation of probes while providing similar biodistribution profiles.

Summarizing, our study aimed to find PNA-based pretargeting probe combinations that balance cost-effective production with effective tumor targeting.

We produced a new set of shorter PNA-based probes for HER2-overexpressing tumors using automated microwave-assisted synthesis. This expanded probe set included three new primary probes (12-mer, 9-mer, and 6-mer) and three secondary probes (8-mer, 7-mer, and 6-mer) in addition to those previously introduced in our prior work.^29^ The primary probes were covalently attached to an anti-HER2 affibody molecule for specific delivery of the primary probe to tumors, while the secondary probes contained a DOTA chelator for radiometal complexing. Through thorough biophysical screening, we identified shorter probe combinations that maintained high-affinity binding, similar to our original Z-HP15:HP16 probe pair.

The new 9-mer Z-HP9 primary probe showed high specificity and affinity for HER2-expressing cells in vitro, and the Z-HP9:[^177^Lu]Lu-HP16 and Z-HP9:[^177^Lu]Lu-HP20 combinations maintained very high-affinity (11 pM) binding between the probe pairs observed for the Z-HP15:[^177^Lu]Lu-HP16 combination in our previous work. In pretargeting studies, we observed no significant differences in tumor-to-kidney ratios between these shorter probe combinations and the original Z-HP15:[^177^Lu]Lu-HP16 pair. The tumor uptake significantly exceeded that in normal tissues. This suggests that using the shorter 9-mer primary probe Z-HP9, in combination with 9-mer HP16 or 8-mer HP20 secondary probes, achieves effective tumor targeting while minimizing radiation to critical organs like the kidneys.

While the pretargeting efficiencies were similar, there is substantial potential for improved production efficiency in transitioning from a 15-base hybridization probe in Z-HP15 to a 9-base probe in Z-HP9. Fewer synthesis steps can result in a more cost-effective production process with reduced reagent and solvent consumption, as well as a simplified and less costly downstream purification process. Additionally, the 9-mer and, notably, the new 8-mer secondary probes exhibit enhanced solubility in aqueous buffers compared to the previously published 15- and 12-mer probes.

## 2 Conclusions

The Z-HP9:HP16 and Z-HP9:HP20 probe combinations show great promise for achieving both cost-effective production and efficient pretargeting in vivo, which increases their potential for translation to clinics.

## 3 Experimental Procedures

### 3.1 Synthesis and Purification of PNA-Based Hybridization Probes

The PNA probes were synthesized using a Biotage Initiator+ Alstra microwave peptide synthesizer with a Rink Amide ChemMatrix resin (0.42 mmol/g) on a 0.1 mmol scale in a 10 mL reactor vial. The synthesis followed a 9-fluorenylmethoxycarbonyl (Fmoc) strategy. Fmoc-protected PNA monomers, Fmoc-PNA-A(Bhoc)–OH, Fmoc-PNA-G(Bhoc)–OH, Fmoc-PNA-C(Bhoc)–OH, and Fmoc-PNA-T–OH, were obtained from PNA Bio (CA, USA). Fmoc-protected amino acids, including Fmoc-Glu(OtBu)–OH, Fmoc-Tyr(OtBu)–OH, and Fmoc-Ser(OtBu)–OH, were sourced from Novabiochem (Germany), Fmoc-Gly-OH from Ambeed (USA), and Fmoc-Lys(Mtt)–OH from IrisBiotech (Germany). The AEEA was purchased from Asta Tech Inc. (USA).

The Fmoc group was removed from each monomer by treatment with dimethylformamide (DMF):piperidine (4:1) for 3 min at room temperature followed by a 10 min treatment. All couplings were conducted for 10 min at 75 °C with Oxyma Pure in DMF and diisopropylcarbodiimide (DIC) in DMF as the coupling reagent. Six equivalents of amino acids and AEEA, with a final concentration of 0.17 M, were used for the coupling steps. In contrast, PNA monomers were dissolved at a final concentration of 0.07 M in DMF, or in DMF:*N*-methyl-2-pyrrolidone (NMP) (1:1) for monomer C, and were used in 4 equiv. The coupling reagents were used with the same number of equivalents as the PNA or amino acid residues. Following each coupling, a capping step was carried out using NMP:2,6-lutidine:acetic anhydride (89:6:5) for 2 min at room temperature.

To optimize the synthesis protocol, a ninhydrin test was performed after each coupling to assess the reaction efficiency. After a positive ninhydrin test, a second coupling was performed to achieve a sufficiently high yield to yield a negative ninhydrin test.

After the synthesis of the complete sequences, a microcleavage was performed by treating a few beads of resin with trifluoroacetic acid (TFA):triisopropylsylane (TIS):Milli-Q water (95:2.5:2.5) for 3 h at room temperature. A 2-(4,7,10-tris(2-(tert-butoxy)-2-oxoethyl)-1,4,7,10-tetraazacyclododecan-1-yl)acetic acid (DOTA-OtBu) was coupled to the N-terminus of the secondary probes 15-mer HP18, 12-mer HP17, 9-mer HP16, 8-mer HP20, 7-mer HP21, and 6-mer HP19. The coupling was performed with 8 equiv of DOTA-OtBu in NMP, 8 equiv of benzotriazol-1-yloxytripyrrolidinophosphonium hexafluorophosphate (PyBOP) in DMF, and 8 equiv of DIEA at room temperature for 1.5 h.

The lysine residue of the primary probes contained an acid-labile 4-methyltrityl (Mtt) side chain protecting group, which was selectively removed by treating with 1% TFA and 5% TIS in dichloromethane (DCM) for 10 × 2 min, or until a ninhydrin test indicated the deprotection of the side chain amino group. DOTA-OtBu was coupled using the same method as for the secondary probes, and another ninhydrin test was performed to assess the coupling efficacy.

All probes were cleaved from the resin for 3 h in TFA:TIS:Milli-Q water (95:2.5:2.5) before precipitation in cold diethyl ether. The precipitates were centrifuged at 4000*g* for 5 min at 4 °C, and the supernatant was discarded. The pellets were resuspended in cold diethyl ether and centrifuged two more times before being dissolved in acetonitrile (ACN) + 0.1% TFA: Milli-Q water +0.1% TFA (1:1), frozen at −80 °C, and freeze-dried overnight.

Freeze-dried crude products of the secondary PNA probes were purified by reversed-phase high-performance liquid chromatography (RP-HPLC) on Zorbax C18 semipreparative columns (300SB-C18, 9.4 × 250 mm^2^, 5 μm pore size; Agilent, Santa Clara, CA, USA). The products were resuspended in 100% buffer A (Milli-Q water +0.1% TFA) and eluted with a gradient of 10–100% buffer B (ACN + 0.1% TFA) over 1 h. Peaks at 260 nm were collected in fractions and analyzed by matrix-assisted laser desorption ionization-time-of-flight mass spectroscopy (MALDI-TOF MS) (4800 MALDI-TOF/TOF, Sciex, Framingham, MA, USA) using an α-cyano-4-hydroxycinnamic acid matrix. HPLC fractions containing the secondary PNA probe were pooled and freeze-dried.

### 3.2 Construction of Expression Plasmids, Protein Expression and Purification

The hybrid expression vector, mpET-45b, was created by merging components from pET-45b(+) and pET-26b(+) vectors. The NcoI site in pET-45b(+) was replaced with NdeI through site-directed mutagenesis. The DNA segment containing the pelB signal sequence and multiple cloning site from pET-26b(+) was amplified and subcloned into the modified pET-45b vector. Gene sequencing by Eurofins Genomics confirmed the mpET-45b vector’s sequence integrity.

To create mpET-45b-Z_HER2:342_-SR-H_6_, the DNA segment encoding Z_HER2:342_-SR-H_6_ was amplified from pAY430-Z_HER2:342_-SR-H_6_^20^ and subcloned into mpET-45b. The final plasmid encodes the Z_HER2:342_-SR-H_6_ protein, with an amino acid sequence that is nearly identical to previous versions used by our group. The only amino acid substitutions (VD to KL) resulted from a change in restriction sites between Z_HER2:342_ and the Sortase A recognition site. The plasmid sequence was verified by sequencing (Eurofins Genomics, Germany).

The DNA sequence for a Ca^2+^-independent Sortase A heptamutant (Srt 7)^51^ was amplified from pASKt15C+SrtA7woH (a kind gift from Teruyuki Nagamune; Addgene plasmid # 65020; http://n2t.net/addgene:65020; RRID:Addgene_65020) and subcloned into the mpET-45b vector to produce a protein with a C-terminal His_6_-tag. A Ca^2+^-independent Sortase A variant with enhanced activity, SrtA 7+,^43^ was generated from the SrtA 7-mpET-45b plasmid through three rounds of site-directed whole plasmid mutagenesis. The final mpET-45b-SrtA7+ plasmid was verified by DNA sequencing (Eurofins Genomics, Germany). Protein expression and purification were done as described elsewhere.^47^ The purity and mass of the proteins after IMAC purification were confirmed using MALDI-TOF MS (see Figures S15–S18).

### 3.3 Conjugation and Purification of PNA-Affibody Constructs

The primary PNA probes (HP15, HP12, HP9, and HP6) were resuspended in HEPES 50 mM, NaCl 150 mM, pH 8 with 10% DMSO, and heated at 95 °C for 5 min. Their concentrations were estimated by measuring absorbance at 260 nm using monomeric PNA-base molar extinction coefficients (A: 13,700, C: 6600, G: 11,700, and T: 8600 M^–1^cm^–1^; monomeric extinction coefficients were obtained from Applied Biosystems).

The conjugation reaction was essentially done as described previously.^20,47^ Briefly, 880 nmol of each primary, unpurified PNA crude product (HP15, HP12, and HP6) was mixed with 440 nmol of Z_HER2:342_-SR-H_6_. The conjugation reaction was initiated by adding SrtA 7+ and incubating the mixture for 15 min at 37 °C. An extra RP-HPLC step was added before HP9 conjugation to remove an unidentified side product.

The reaction mixture was then processed using a Ni-NTA HisPur (Thermo Scientific) column. Nonreacted and His_6_-tagged affibody was allowed to bind to the resin, followed by elution with imidazole. SDS-PAGE and RP-HPLC were used for monitoring and purification. Example of an SDS–PAGE gel from the conjugation of HP12 to Z_HER2:342_-SR-H_6_ can be seen in Figure S13. The mass of PNA-affibody conjugates was verified using ESI-Q-TOF-MS (Impact II, Bruker Daltonics, Billerica, MA, USA).

The final purity of Z-HP15, Z-HP12, Z-HP9, HP16, and HP20 was analyzed using analytical RP-HPLC (300SB-C18, 4.6 × 150 mm2, 3.5 μm pore size; Agilent, Santa Clara, CA, USA) with a gradient of 0–100% buffer B in buffer A over 1 h. The purities were determined by comparing the integrated areas under the peaks in the HPLC elution profile at 260 nm. All compounds are >95% pure by HPLC analysis.

### 3.4 Kinetic Characterization of PNA Probe Binding Using SPR

We utilized SPR on a Biacore T200 instrument (Cytiva) to examine how secondary PNA probes (6-mer HP19, 7-mer HP21, 8-mer HP20, and 9-mer HP16) bind to primary agents. These experiments were conducted using dextran-coated CM5 sensor chips and PBS-T buffer at pH 7.4. The ligands were immobilized through a standard EDC/NHS coupling method, and the chip surface was sealed with ethanolamine. A reference surface with no primary agent was also included.

For ranking the off-rate (*k*_d_) of the secondary probes, we employed single-cycle kinetics (SCK) at 25 °C with a flow rate of 50 μL/min. The primary probes (Z-HP15, Z-HP12, and Z-HP9) were immobilized at 160 RU, 200 RU, and 200 RU, respectively. Secondary PNA probes were injected at various concentrations, and dissociation was monitored for 10,000 s. To regenerate the chip surface, we used two 30 s injections of 10 mM glycine-HCl, pH 1.5.

We also conducted MCK to gain a more detailed understanding of how HP16 and HP20 bind to Z-HP9, and compared HP16 binding to Z-HP15, at both 25 and 37 °C. Z-HP15 (310 RU) and Z-HP9 (450 RU) were immobilized on a CM5 chip, and we injected various concentrations of HP16 and HP20 (1.4, 7, 14, and 70 nM) at a flow rate of 30 μL/min. Association and dissociation times were set at 700 and 1800 s, respectively, with regeneration accomplished through a 30 s injection of 10 mM glycine-HCl, pH 1.5.

All SPR experiments were performed in duplicate, and data analysis was conducted using the Biacore T200 Evaluation software version 2.0 with a 1:1 binding model.

### 3.5 Materials Radiolabeling

Most of the chemicals used in this study were purchased from Sigma-Aldrich, Sweden AB. The buffers used for labeling were prepared using high-quality Milli-Q water and purified from metal contamination using Chelex 100 resin (Bio-Rad Laboratories, USA). No-carrier-added ^177^LuCl_3_ was purchased from PerkinElmer (Waltham, MA, USA). The NAP-5 size-exclusion columns used for purification were purchased from Cytiva. Radioactivity was measured using an automated gamma spectrometer with a NaI (TI) detector (2480 Wizard, Wallac, Finland). A Cyclone Storage Phosphor System and OptiQuant image analysis software (PerkinElmer, Waltham, MA, USA) were used for radioactivity distribution on an instant thin layer chromatography (iTLC) strip measurement.

In vitro cell studies were performed using HER2-expressing ovarian cancer SKOV-3 and breast cancer BT-474 cells from the American Type Culture Collection (ATCC, Manassas, MA, USA). Cells were cultured in Roswell Park Memorial Institute (RPMI) 1640 medium (Sigma-Aldrich), supplemented with 10% (for SKOV-3) or 20% (for BT-474) fetal bovine serum (FBS), 2 mM l-glutamine, 100 IU/mL penicillin, and 100 mg/mL streptomycin. These media are referred to as complete media in the text. Cells were seeded in cell culture dishes with 10^6^ cells per dish. Data on in vitro studies were analyzed by an unpaired 2-tailed *t* test using GraphPad Prism (version 9.0000 for Windows; GraphPad Software LLC, San Diego, CA, USA) to determine significant differences (*p* < 0.05). To determine significant differences (*p* < 0.05) in vivo, data on biodistribution were analyzed by ANOVA using GraphPad Prism (version 6 for Windows; GraphPad Software)

### 3.6 Radiolabeling of Probes with ^177^Lu and In Vitro Stability

Radiolabeling of primary and secondary probes with ^177^Lu was performed using a previously described method.^29^ Briefly, 100 μL of 0.2 M of NH_4_Ac, pH 5.5 was added to 30 μg of the probe (100 μL of 0.3 mg/mL stock in NH_4_Ac) to provide right pH for the labeling. The mixture was heated at 95 °C for 10 min, followed by sonication for 5 min, and reheating at 95 °C for 10 min to ensure complete dissolving. A predetermined amount of ^177^Lu (60 MBq) was added, and the labeling mixture was incubated at 95 °C for 1 h. RCY was then analyzed using iTLC developed with 0.2 M citric acid, pH 2.0. If necessary, the purification step was performed using NAP-5 column size-exclusion purification on the NAP-5 column, pre-equilibrated and eluted with 1% BSA in PBS. To cross-validate radio-iTLC data further, reverse phase-HPLC was conducted on an Elite LaChrom system (Hitachi, VWR, Darmstadt, Germany) consisting of an L-2130 pump, a UV detector (L-2400), and a radiation flow detector (Bioscan, Washington, DC, USA) coupled in series. Purity analysis of the radiolabeled compound was performed using an analytical column (Vydac RP C18 column, 300 Å; 3 × 150 mm; 5 μm). HPLC conditions were as follows: *A* = 10 mM TFA/H_2_O, *B* = 10 mM TFA/acetonitrile, UV-detection at 220 nm, gradient elution: 0–15 min at 5–70% B, 15–18 min at 70–95% B, 19–20 min at 5% B, and a flow rate was 1.0 mL/min. To evaluate the stability of the labeled conjugates, a fraction of freshly radiolabeled conjugate (10 μL, 0.4 μg) was incubated with a 500-fold molar excess of EDTA at 37 °C for 1 h. Incubation was also performed in PBS as a control. Samples were run in triplicates and analyzed by radio-iTLC.

### 3.7 In Vitro Studies

To evaluate the binding affinity of the radiolabeled conjugates to HER2 receptors and the cell-bound primary probe, their binding and dissociation kinetics from SKOV-3 cells were measured using a LigandTracer yellow instrument (Ridgeview Instruments AB, Vänge, Sweden) as described previously.^52^ Cells were seeded on a local area of a cell culture dish (89 mm in diameter, Nunclon, NUNC A/S, Roskilde, Denmark) 1 day before the experiment (3 × 10^6^/dish). The measurements were performed at room temperature to prevent internalization. The SKOV-3 cells were presaturated with the primary agent (1 nM) for 2 h. Thereafter, the media were washed three times to remove the unbound primary agent. After adding the fresh nonlabeled media, the secondary probes were added to obtain concentrations of 1 and then 5 nM. After certain time incubation in the presence of labeled conjugate, the radioactive medium was replaced with fresh nonradioactive medium, and the dissociation curve was recorded for several hours. Additionally, to evaluate the binding affinity of primary agents, the experiment was performed by adding [^177^Lu]Lu-Z-HP9 and [^177^Lu]Lu-Z-HP12 (180, 540 pM, and 1.62 nM). After that, cells were treated as mentioned above. The data were analyzed by InteractionMap software (Ridgeview Instruments AB, Uppsala, Sweden) to calculate the association, dissociation rates, and dissociation constant at equilibrium (*K*_D_) for each radioconjugate. The analysis was done in duplicates.

In vitro binding specificity of the primary probes ([^177^Lu]Lu-Z-HP9 and [^177^Lu]Lu-Z-HP12) to HER2-expressing cells was evaluated by saturation of HER2 receptors with an excess of nonlabeled anti-HER2 affibody molecule (500 nM) in the blocked group for 30 min at room temperature. For the nonblocked group, the same volume of complete media was added. Then, both groups were incubated with a radiolabeled primary probe (1 nM) in a humidified incubator (5% CO_2_, 37 °C) for 1 h. After incubation, the cells were washed with 2 mL media and treated with 0.5 mL of trypsin-EDTA for 15 min at 37 °C. Once the cells were detached, they were collected with 0.5 mL of complete media. Radioactivity of cells was measured using an automatic gamma spectrometer, and the cell-associated radioactivity was calculated.

Pretargeting specificity was also tested in four sets of dishes to evaluate in vitro pretargeting specificity; to show in vitro pretargeting, cells were incubated with nonlabeled primary agent Z-HP9 (1 nM) for 1 h at 37 °C and washed. Thereafter, ^177^Lu-secondary probe (10 nM) was added, and cells were incubated for 1 h at 37 °C. After that, cells were treated as mentioned above. To show that if the pretargeting is HER2-mediated, cells were presaturated with an excess amount of anti-HER2 affibody molecule (1000 nM) for 5 min at 37 °C to saturate HER2 receptors prior to adding nonlabeled Z-HP9. Then, a radiolabeled secondary probe (10 nM) was added and incubated for 1 h at 37 °C. Further treatment was the same as pretargeting group. To demonstrate that the pretargeting is PNA-mediated, cells were incubated with an excess amount of nonlabeled secondary probe (300 nM) for 1 h at 37 °C prior to adding the radiolabeled secondary probe (10 nM) and incubated for 1 h at 37 °C. Further treatment was the same as mentioned above. To evaluate unspecific binding, the radiolabeled secondary probe (10 nM) was added to the cells without any pretreatment with the primary agent and incubated for 1 h at 37 °C. After incubation, the cells were washed and treated as mentioned above.

A validated acid-wash method^53^ was used for cellular processing and retention of radiolabeled probes by SKOV-3 and BT-474 using interrupted incubation procedure. Cells were incubated with the primary agent (1 nM) for 1 h at 4 °C. Then, the media was removed, and the cells were washed. Thereafter, a radiolabeled secondary probe (10 nM) was added and incubated for 30 min at 4 °C. After that, the media was removed, and the cells were washed again prior to the addition of new complete media. The cells were then incubated at 37 °C in a humidified incubator for 1, 2, 4, 8, and 24 h. At each designated time points, a set of dishes was taken out from the incubator, and the media was collected. The cells were washed with cold serum-free media (2 mL) and treated with 0.2 M glycine buffer containing 4 M urea, pH 2.0 (1 mL) for exact 5 min on ice to evaluate membrane fraction. The acidic solution was collected, and the cells were additionally washed with the same buffer (1 mL) and collected. To evaluate the internalized fraction, the cells were then treated with 1 M NaOH (1 mL), incubated for 30 min at 37 °C, and collected. The cells were also additionally washed with the same buffer (1 mL) and collected. The radioactivity in each fraction was measured using an automated gamma spectrometer. The radioactivity of acidic and alkaline fractions was considered as membrane and internalized parts, respectively. The cellular processing and retention of radiolabeled primary agents were performed as described above using 1 nM concentration of the primary agent.

### 3.8 In Vivo Studies

Animal studies were planned in agreement with EU Directive 2010/63/EU for animal experiments and Swedish national legislation concerning the protection of laboratory animals and were approved by the Ethics Committee for Animal Research in Uppsala, Sweden (animal permission C4/16). HER2-positive xenografts were implanted by subcutaneous injection of 10^7^ SKOV-3 cells in the right hind legs of female BALB/C nu/nu mice. Three weeks after implantation, mice were randomized into 8 groups, with 4 mice in each group. At the time of the experiment, the average animal weight was 20 ± 1 g. The average tumor weight was 0.26 ± 0.14 g. The mice were euthanized at predetermined time points by overdose of Ketamine/Xylazine anesthesia, followed by heart puncture. The organs of interest such as blood, lung, liver, spleen, kidney, muscle, bone, and tumor were collected, weighed, and subsequently their radioactivity was measured. Organs and tumor uptake were calculated as the percentage of the injected dose per gram of the sample (%ID/g).

The PNA pretargeting method used in this study was following the previously optimized study^26^ at two time points, 4 and 144 h after injection. Four groups of mice were injected with the new primary agent Z-HP9 (45 μg, 4 nmol in 100 μL PBS in each mouse). After 16 h, [^177^Lu]Lu-HP16 and [^177^Lu]Lu-HP20 (194 pmol in 100 μL 2% BSA in PBS, 170 kBq) were injected intravenously. At 4 and 144 h postinjection, the mice were euthanized and subsequently treated as described above. As comparison, a similar method was performed for the biodistribution of [^177^Lu]Lu-HP16 (194 pmol in 100 μL 2% BSA in PBS, 170 kBq) at two time points (4 and 144 h p.i.) on 2 groups of mice that were injected with second-generation Z-HP15 as the primary agent (50 μg, 4 nmol in 100 μL PBS per mouse).^29^

To evaluate in vivo specificity, 2 groups of mice were injected with [^177^Lu]Lu-HP16 and [^177^Lu]Lu-HP20 without preinjection of the primary agent. At 4 h postinjection, the mice were euthanized and treated as mentioned above.

## Data Availability

Data will be made available on request.

## Supporting Information Available

HPLC, MALDI-TOF, SDS-PAGE, ESI-TOF, solubility data, CD data, SPR, UV-melt data, and LigandTracer (PDF)
