# Supplementary material for: Shorter Peptide Nucleic Acid Probes Improve Affibody-Mediated Peptide Nucleic Acid-Based Pretargeting
Source: ACS Pharmacol Transl Sci. 2024 Apr 29;7(5):1595–611. doi: 10.1021/acsptsci.4c00106 (PMC11091976; doi:10.1021/acsptsci.4c00106)
Supplement: Supplementary file 1 — pt4c00106_si_001.pdf [file pt4c00106_si_001.pdf]

Supplementary data

Shorter peptide nucleic acid probes improve Affibody-mediated peptide nucleic acid-based pretargeting

Kristina Westerlund[1§], Maryam Oroujeni[2,3§], Maxime Gestin[1], Jacob Clinton[1], Alia Hani Rosly[2], Hanna Tano[1], Anzhelika Vorobyeva[2], Anna Orlova[4], Amelie Eriksson Karlström[1*] and Vladimir Tolmachev[2]

[§]Contributed equally to this study

[1] Department of Protein Science, School of Engineering Sciences in Chemistry, Biotechnology and Health, KTH Royal Institute of Technology, 106 91 Stockholm, Sweden

[2]Department of Immunology, Genetics and Pathology, Uppsala University

[3]Affibody AB, 171 65 Solna, Sweden

[4]Department of Medicinal Chemistry, Uppsala University, 751 23 Uppsala, Sweden;

*Corresponding author
E-mail address: ameliek@kth.se
Full postal address: Department of Protein Science, School of Engineering Sciences in Chemistry, Biotechnology and Health, KTH Royal Institute of Technology, AlbaNova University Center, 106 91 Stockholm, Sweden

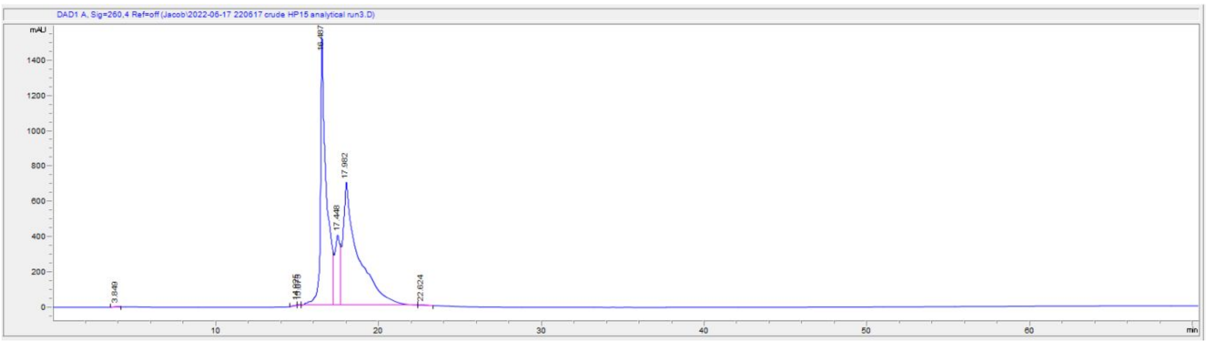

Figure S1: HPLC chromatogram of the crude products from the synthesis of the primary PNA probe 15-mer HP15. 16.5 min: correct product. 17.5 min and 18 min: unidentified by-products.

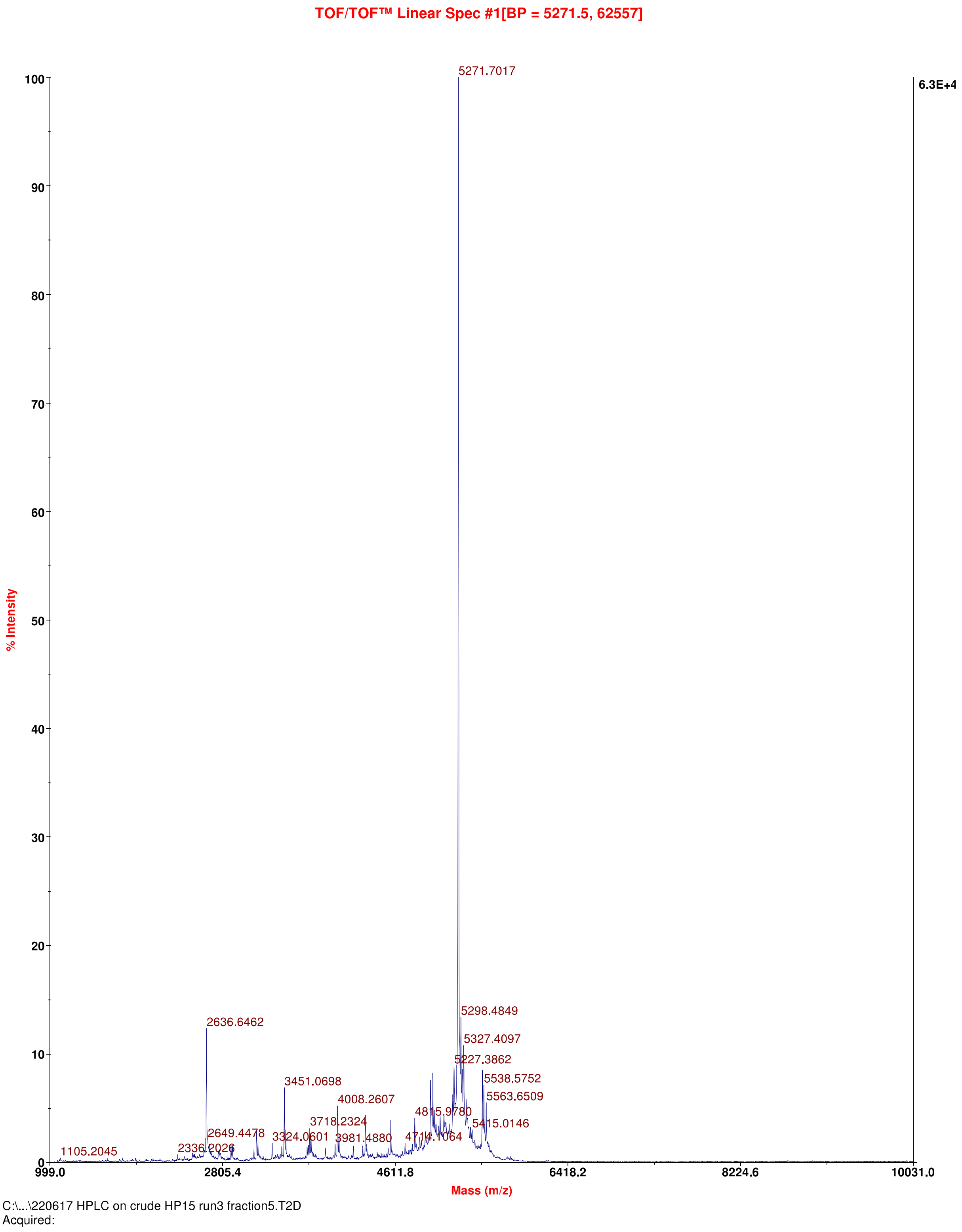

Figure S2: MALDI-TOF mass spectrum of HP15. Detected mass is 5272 Da, while theoretical mass is 5274 Da.

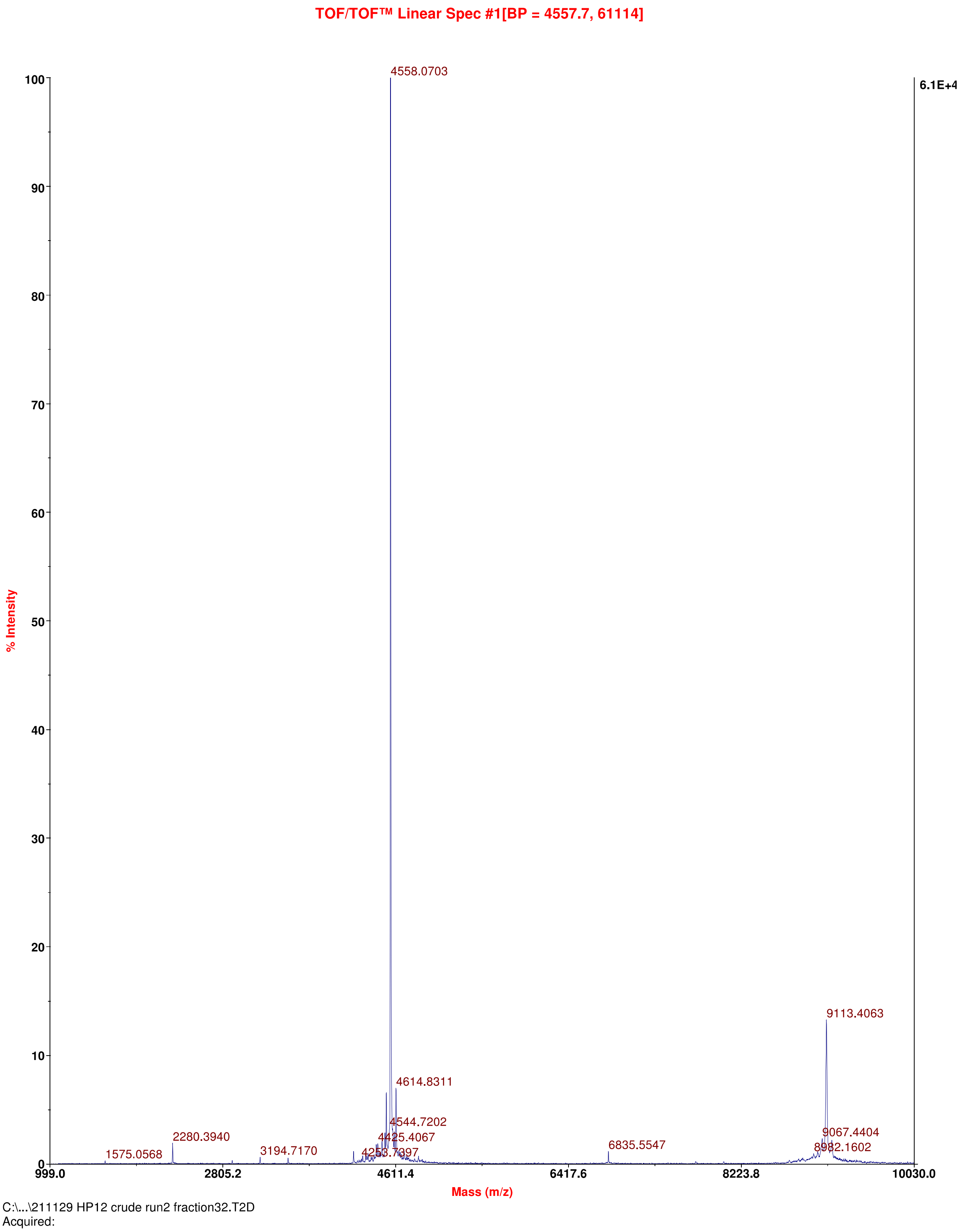

Figure S3: MALDI-TOF mass spectrum of HP12. Detected mass is 4558 Da, while theoretical mass is 4556 Da.

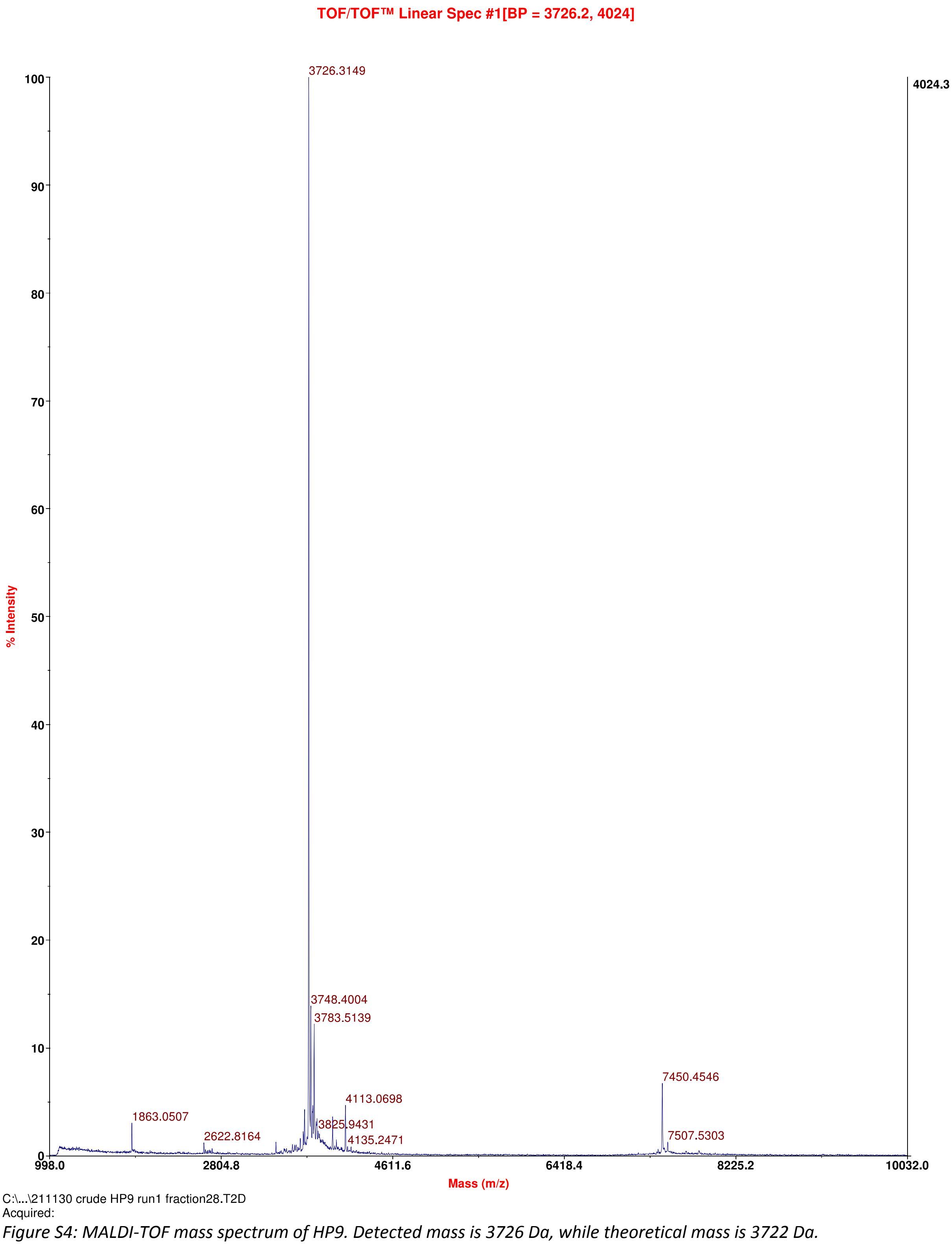

Figure S4: MALDI-TOF mass spectrum of HP9. Detected mass is 3726 Da, while theoretical mass is 3722 Da.

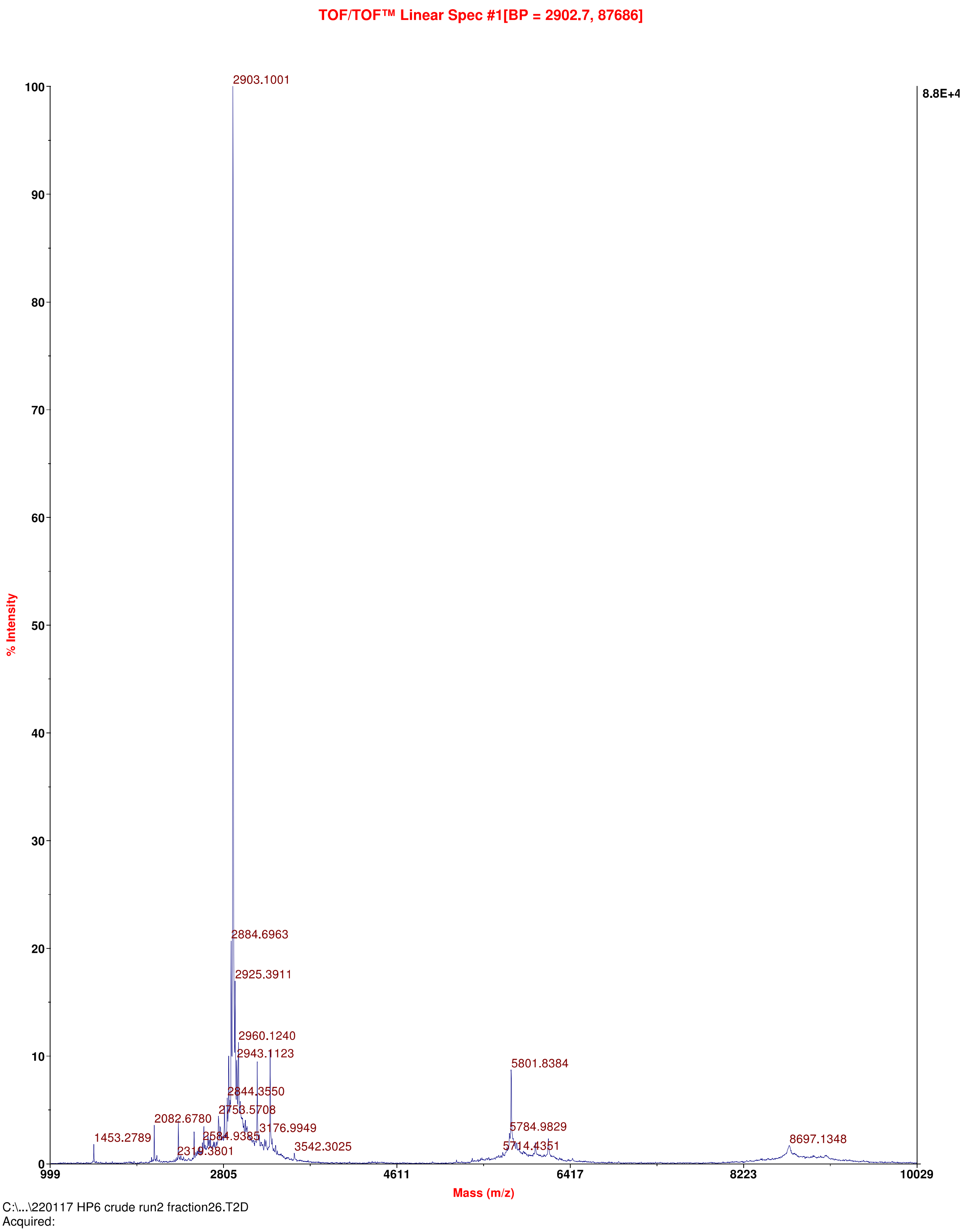

Figure S5: MALDI-TOF mass spectrum of HP6. Detected mass is 2903 Da, while theoretical mass is 2899 Da.

A

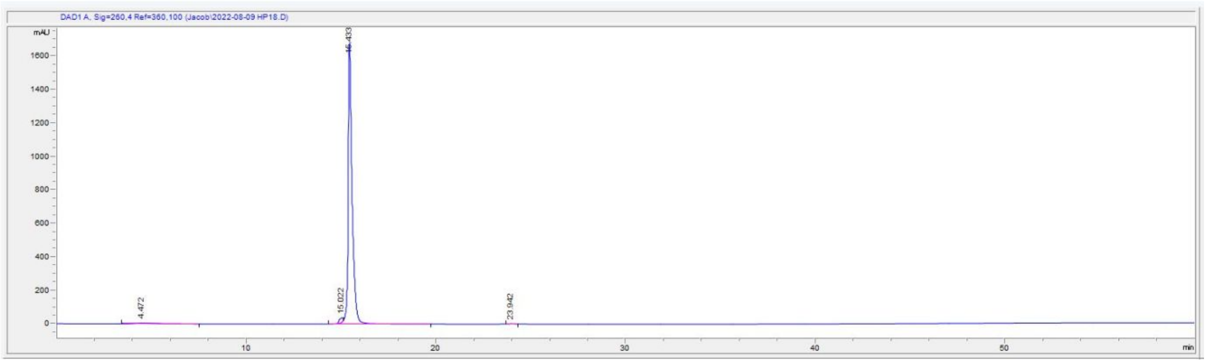

B

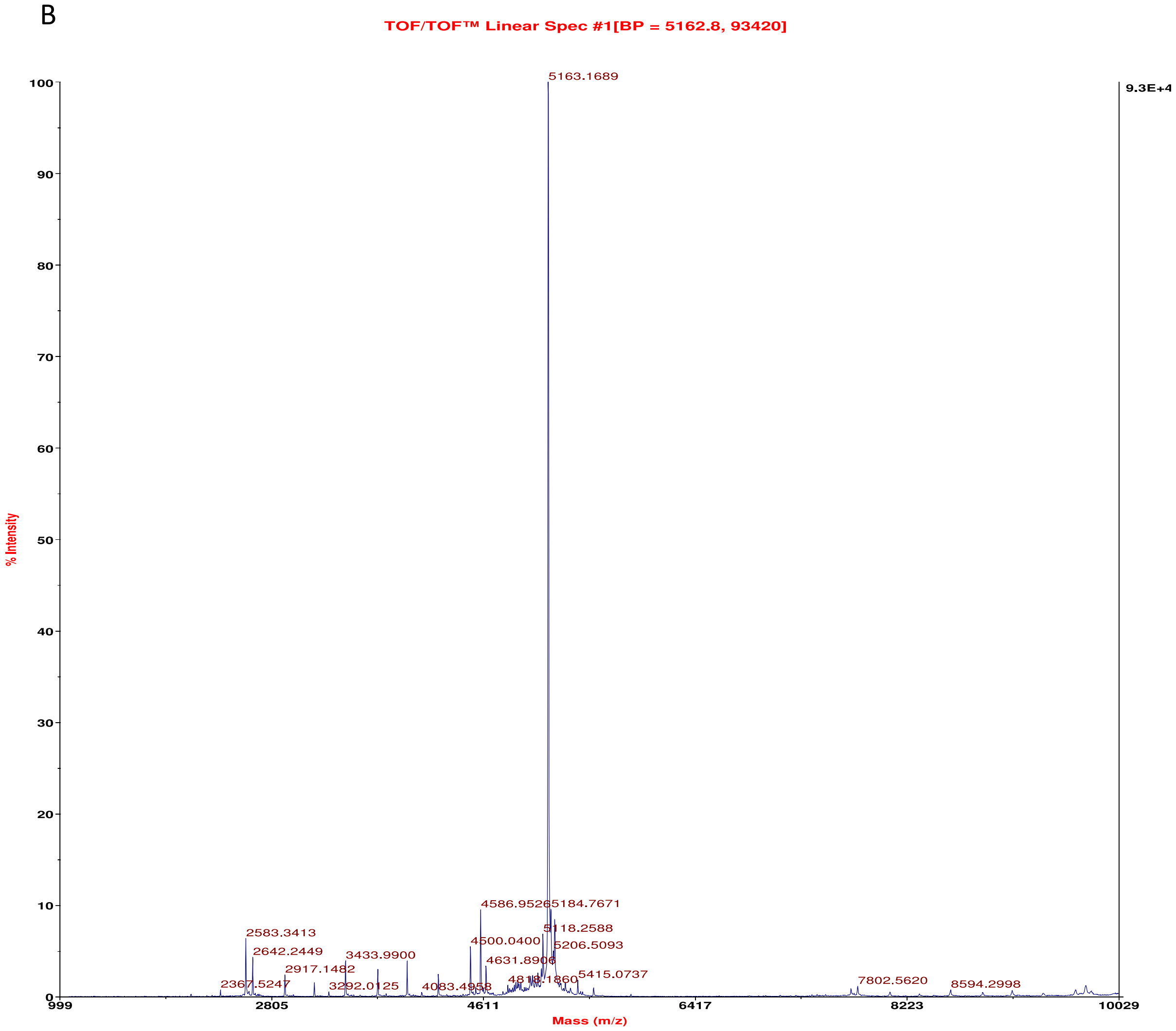

Figure S6: (A) Analytical HPLC of the secondary PNA probe 15-mer HP18 (B) MALDI-TOF mass spectrum of HP18. Detected mass is 5163 Da, while theoretical mass is 5168 Da.

A

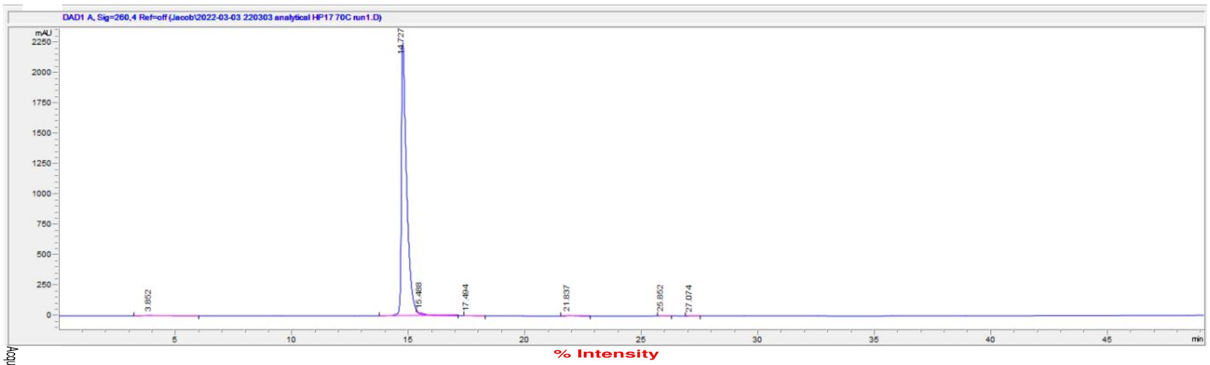

B

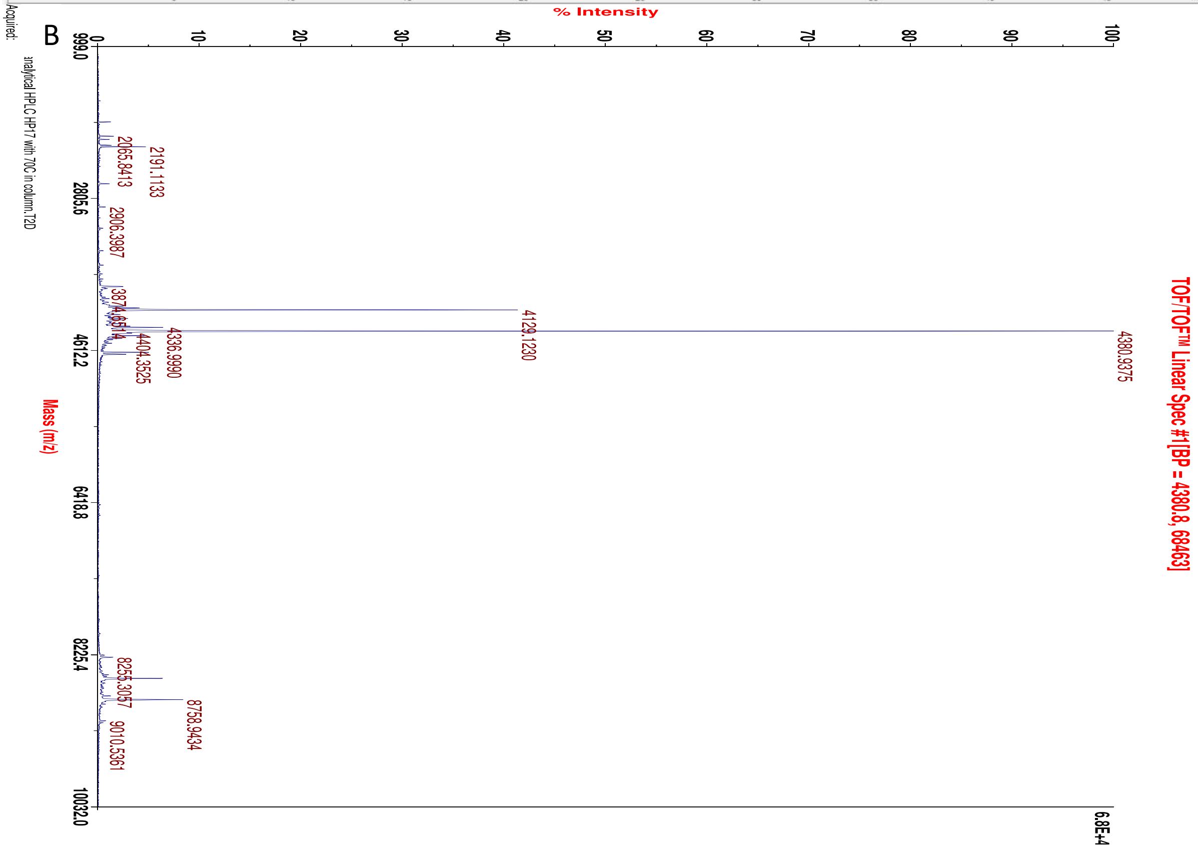

Figure S7: (A) Analytical HPLC of the secondary PNA probe 12-mer HP17. (B) MALDI-TOF mass spectrum of HP17. Detected mass is 4381 Da, while theoretical mass is 4375 Da.

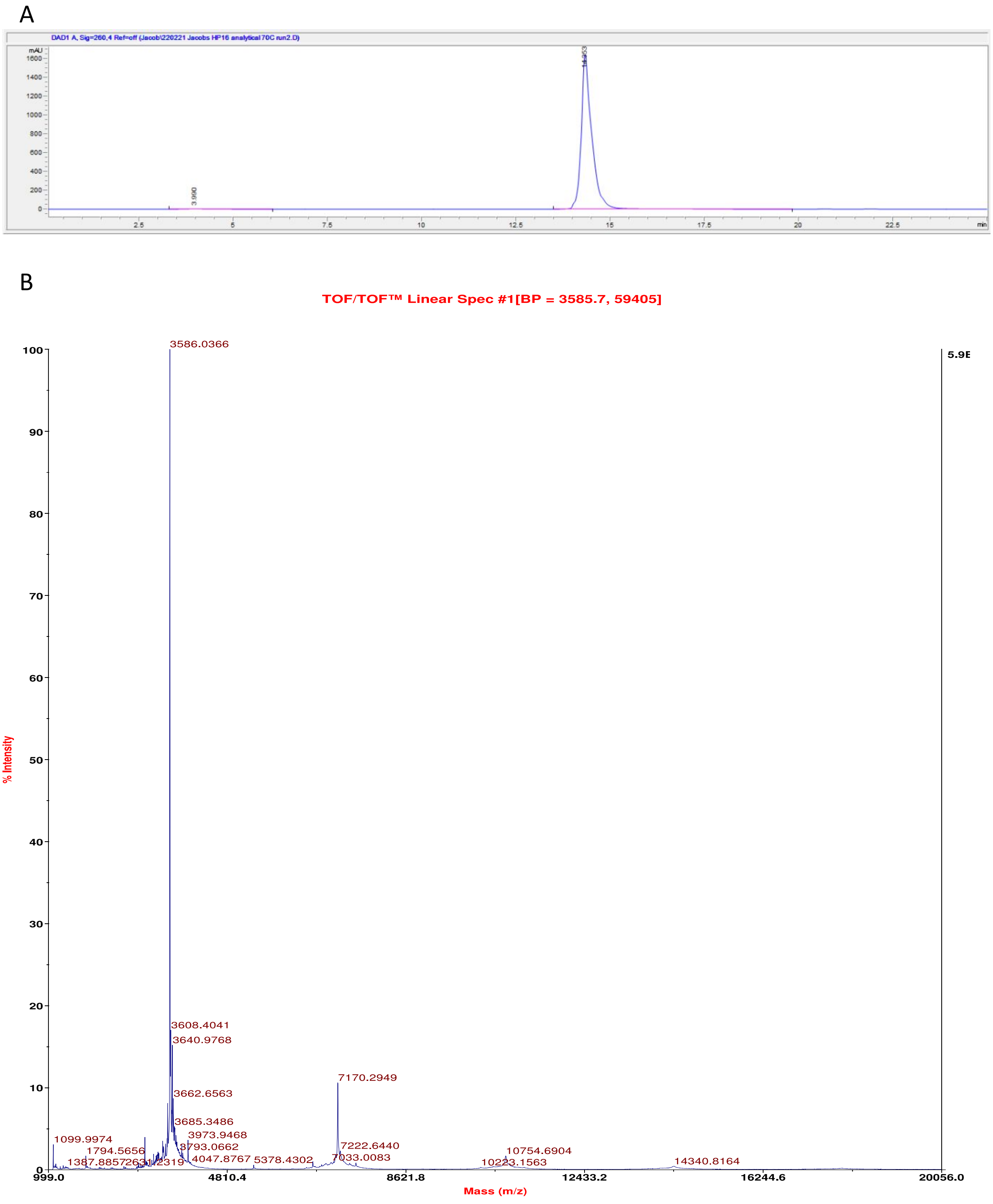

Figure S8: (A) Analytical HPLC of the secondary PNA probe 9-mer HP16. (B) MALDI-TOF mass spectrum of HP16, showing experimental mass of 3586 Da. The theoretical mass is 3583 Da.

A

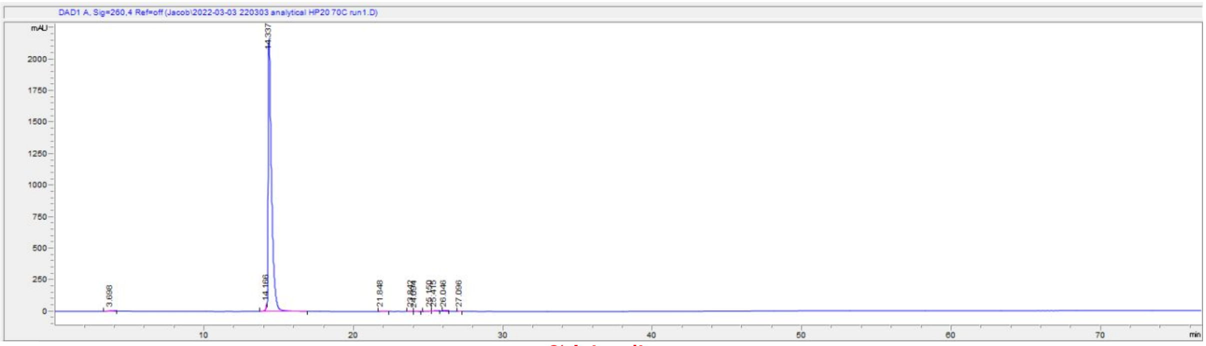

B

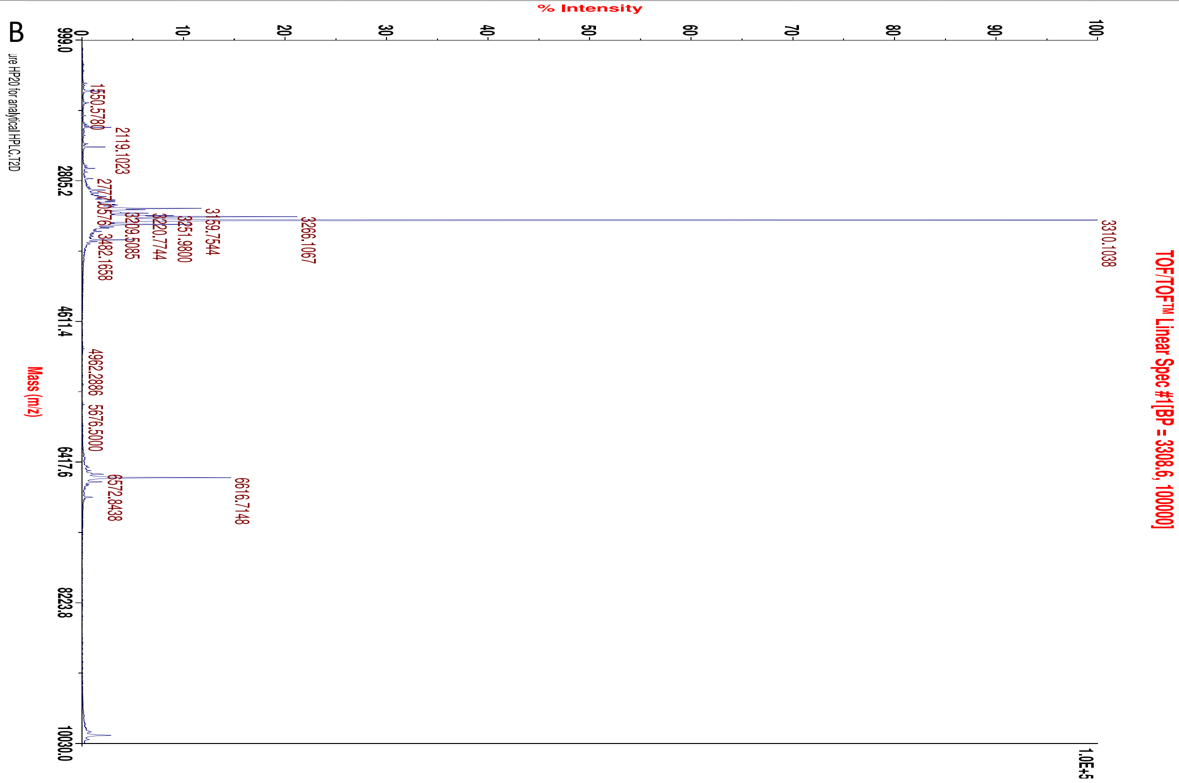

Figure S9: (A) Analytical HPLC of the secondary PNA probe 8-mer HP20. (B) MALDI-TOF mass spectrum of HP20. Detected mass is 3310 Da, and theoretical mass is 3307.

A

B

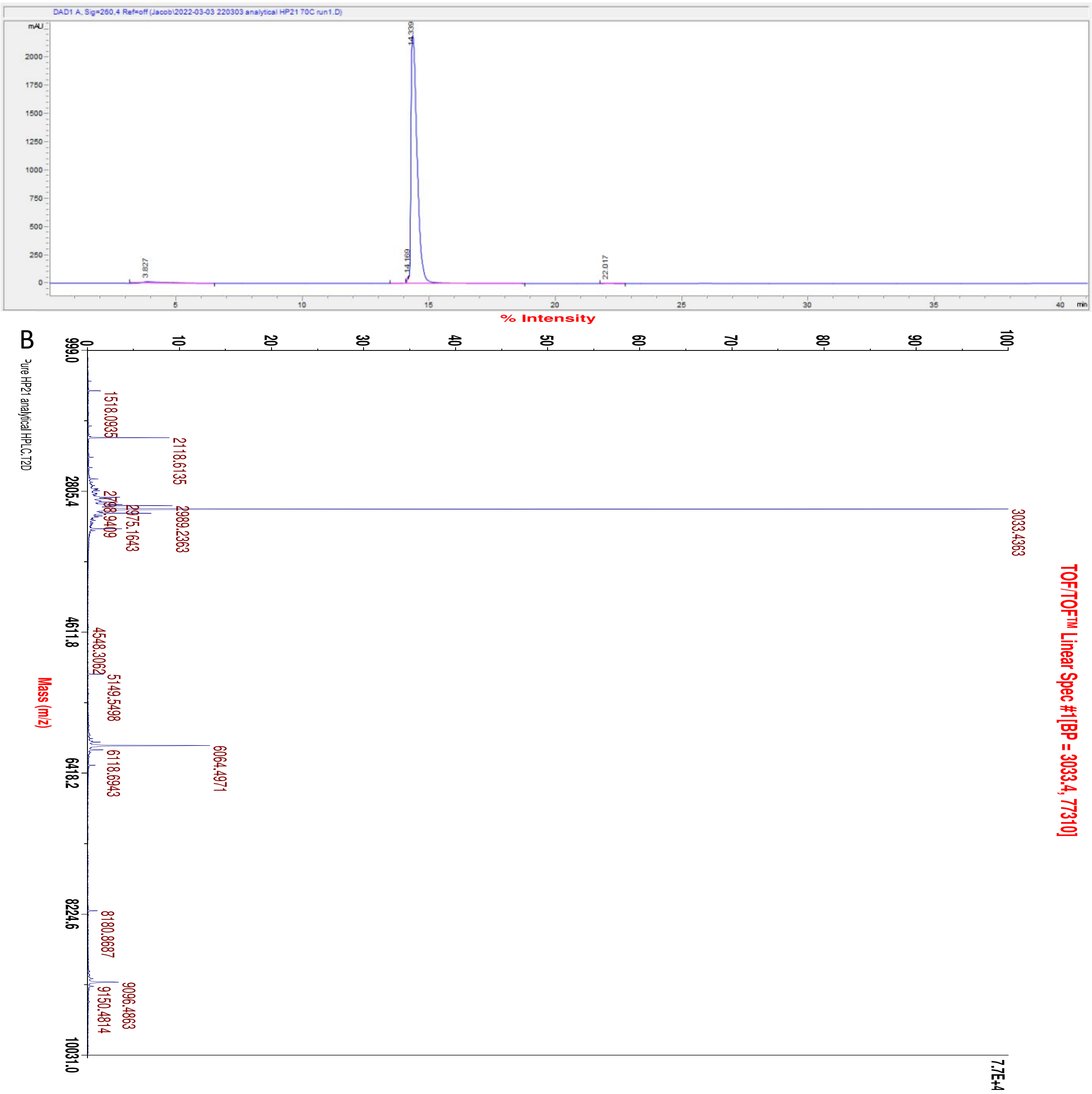

Figure S10: (A) Analytical HPLC of the secondary PNA probe 7-mer HP21. (B) MALDI-TOF mass spectrum of HP21, showing detectedl mass of 3033 Da. The theoretical mass is 3032 Da.

A

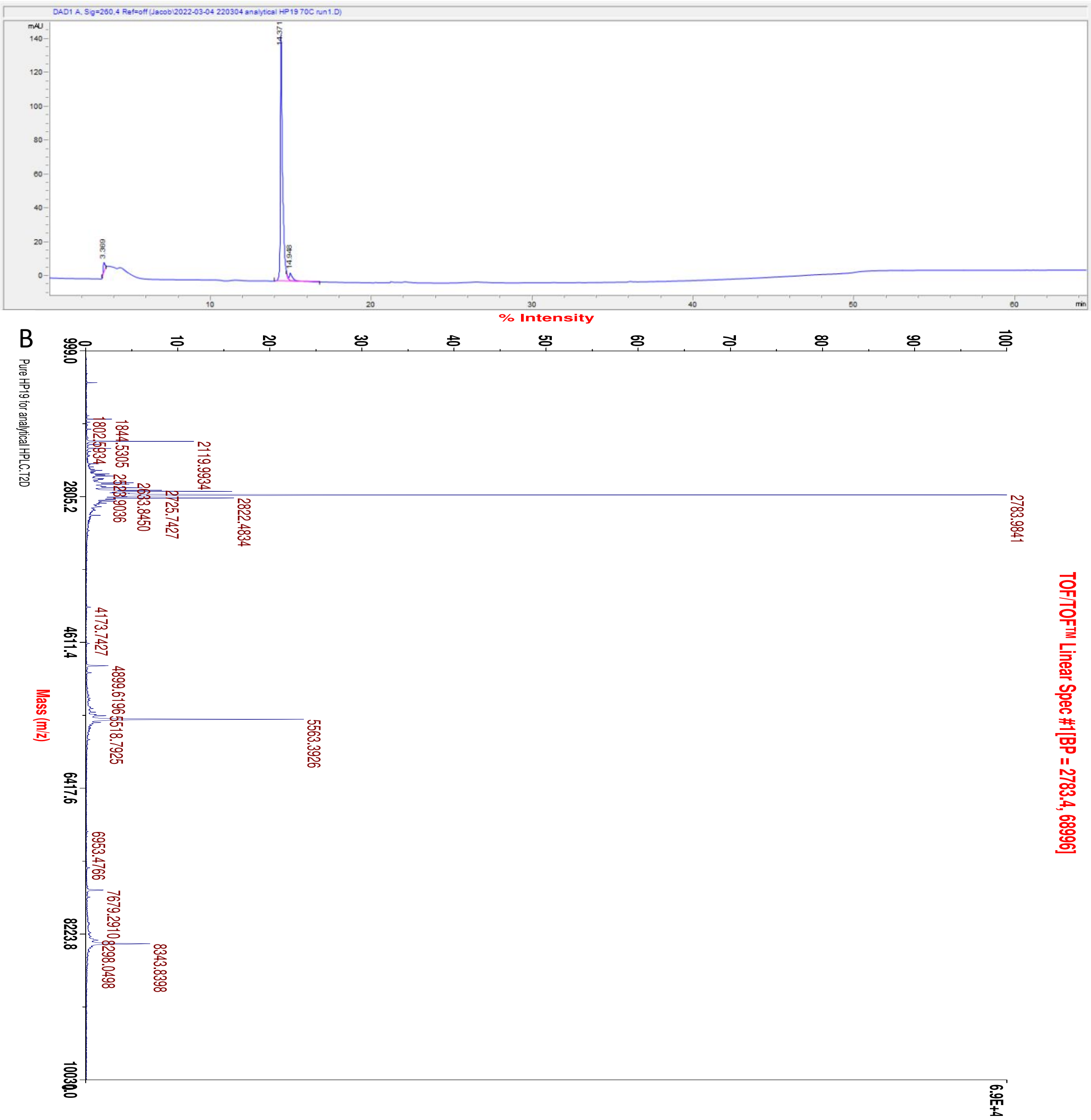

Figure S11: (A) Analytical HPLC of the secondary PNA probe 6-mer HP19. (B) MALDI TOF mass spectrum of HP19, showing the detected mass of 2784 Da. The theoretical mass is 2780 Da.

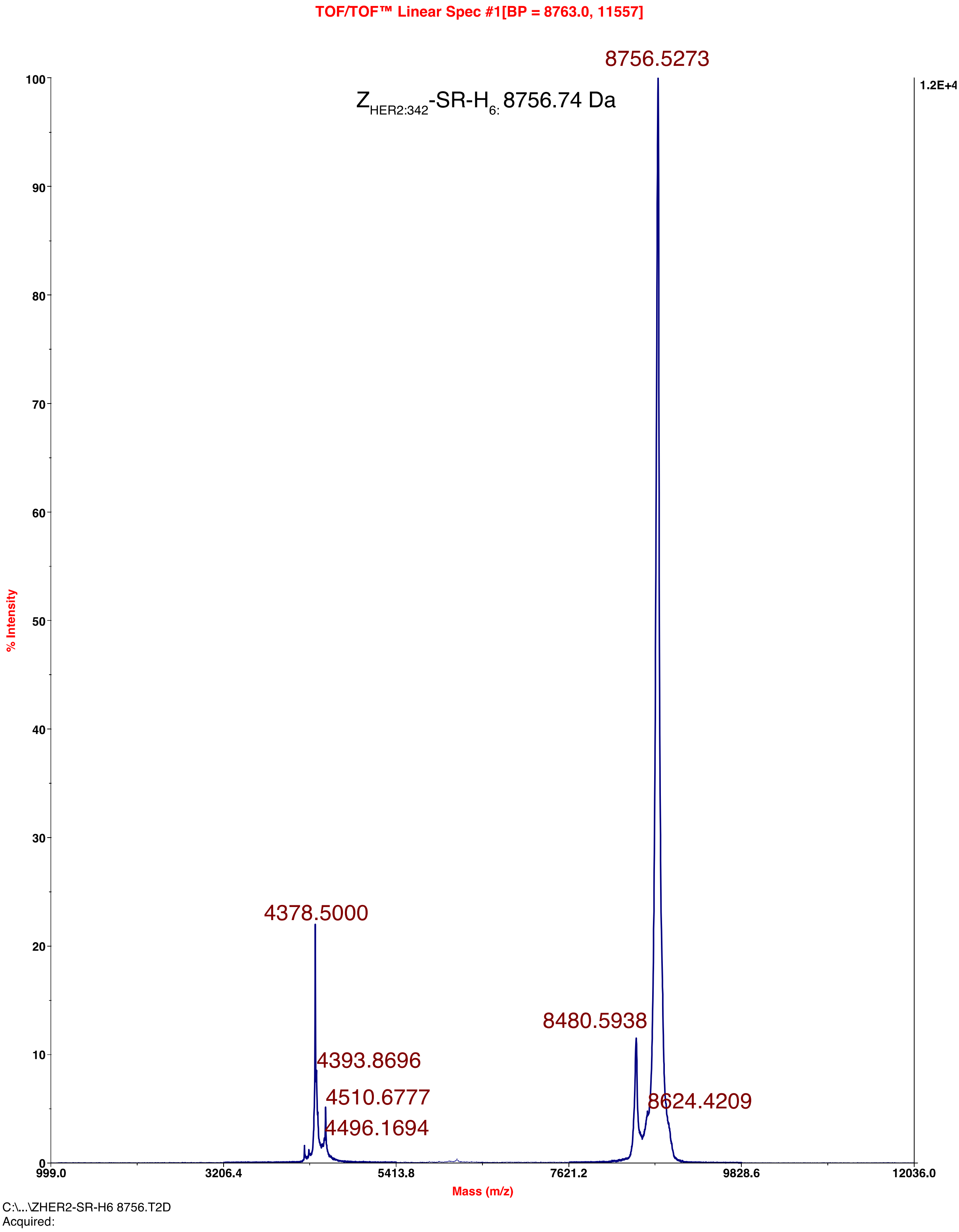

Figure S12: MALDI-TOF mass spectrum of the affibody $Z_{HER2:342}$-SR-H_6 after IMAC purification.

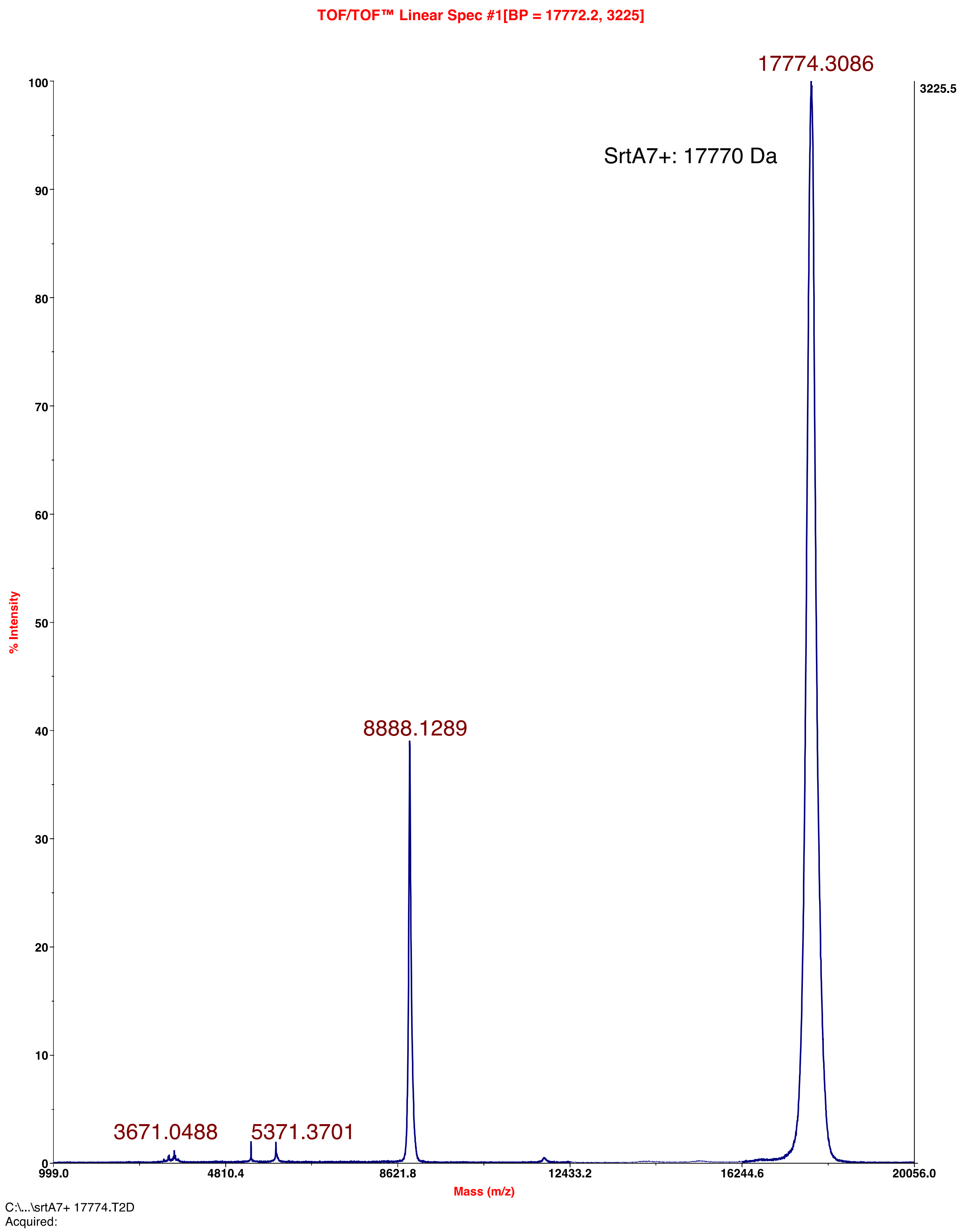

Figure S13: MALDI-TOF mass spectrum of the enzyme SrtA7+ after IMAC purification.

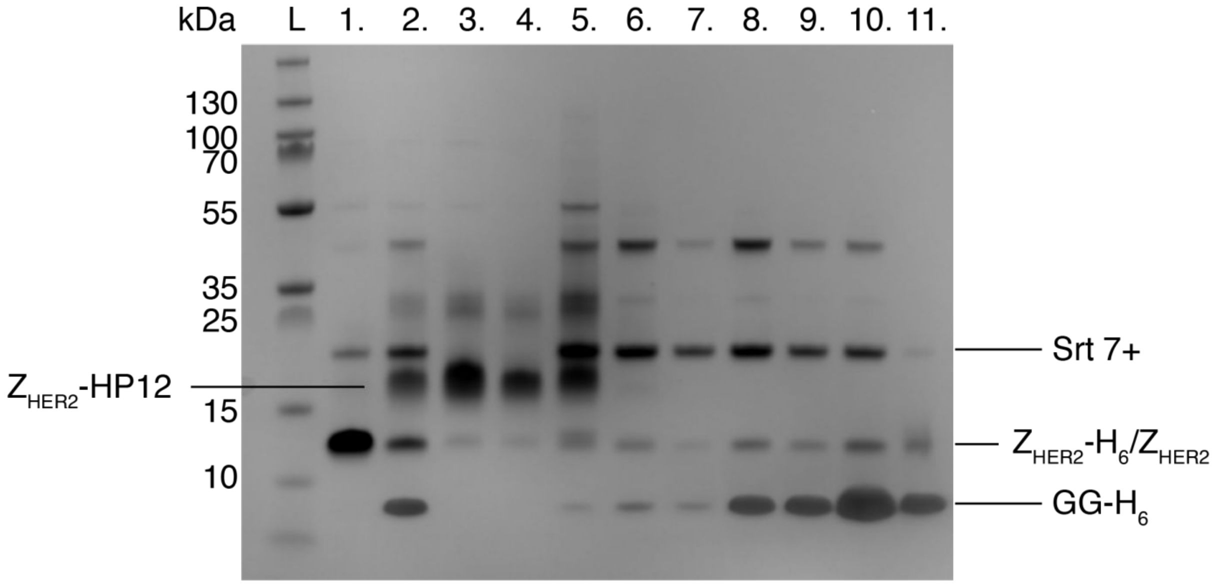

Figure S14: SDS-PAGE analysis of Z_{HER2}-HP12 conjugation and subsequent IMAC purification. L: Molecular weight ladder. Lane 1: Conjugation mixture at the start of the conjugation. Lane 2: Conjugation mixture after a 15-minute conjugation reaction. Lane 3: IMAC flow through. Lane 4: Wash step. Lane 5-7: Elution with 50 mM imidazole. Lane 8-9: Elution with 100 mM imidazole. Lane 10: Elution with 250 mM imidazole. Lane 11: Elution with 1M imidazole. Fractions in lanes 3-6 contain the Z_{HER2}-HP12 conjugate and were pooled and further purified using RP-HPLC.

A

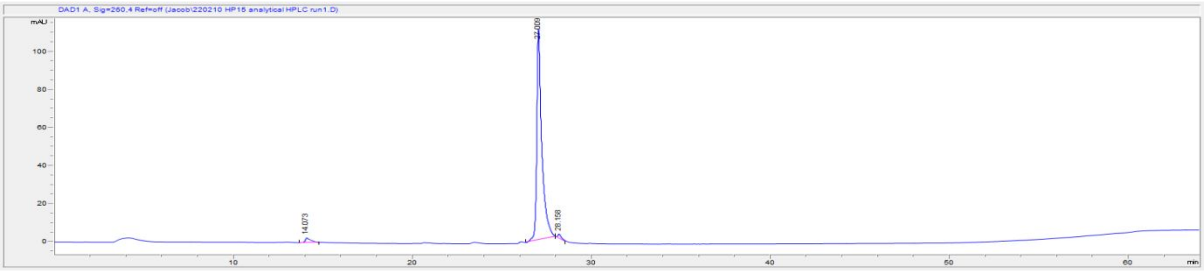

B

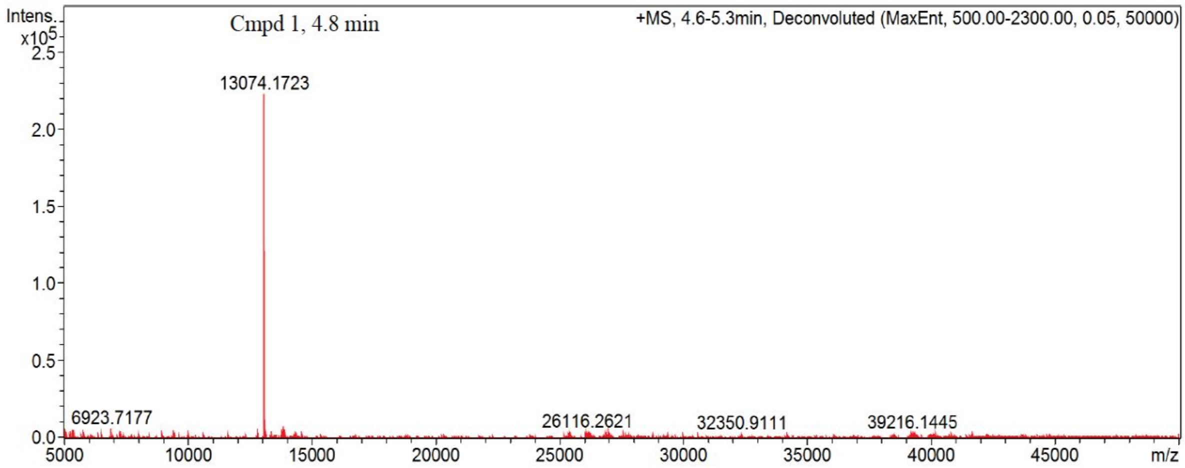

Figure S15: (A) Analytical HPLC of Z_{HER2}-HP15. (B) ESI-TOF mass spectrum of Z_{HER2}-HP15. Theoretical Mass: 13076 Da.

A

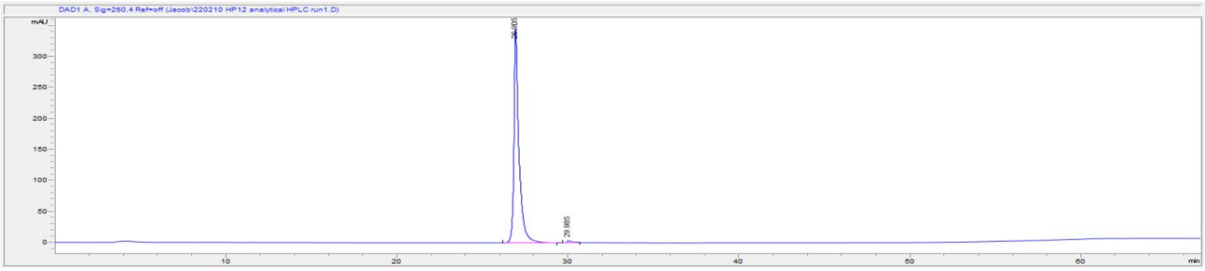

B

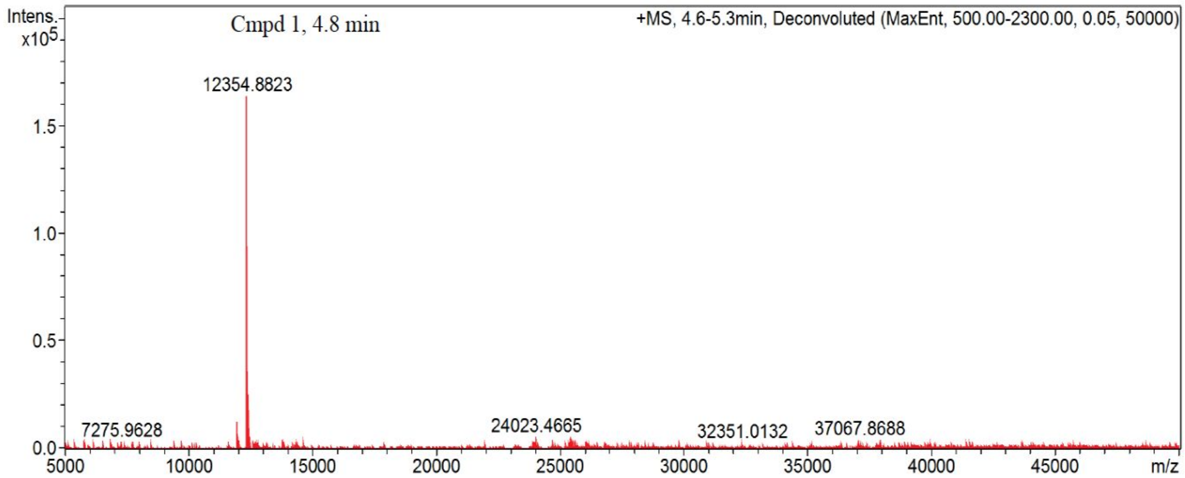

Figure S16: (A) Analytical HPLC of Z_{HER2}-HP12. (B) ESI-TOF mass spectrum of Z_{HER2}-HP12. Theoretical Mass: 12357 Da.

A

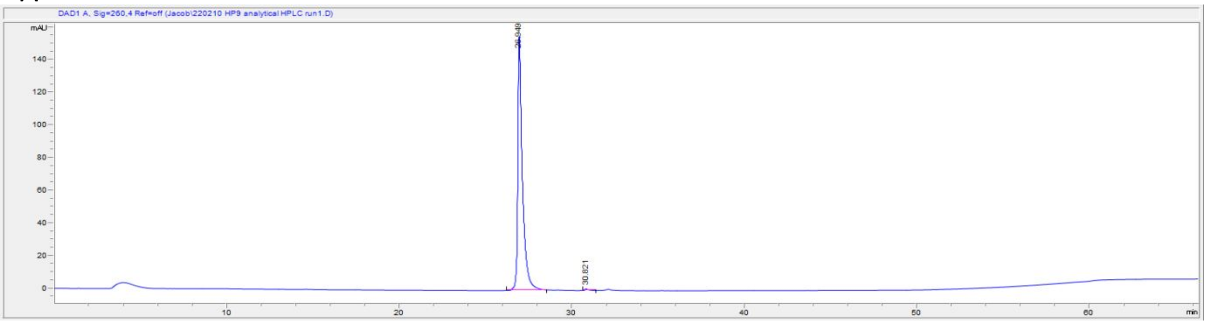

B

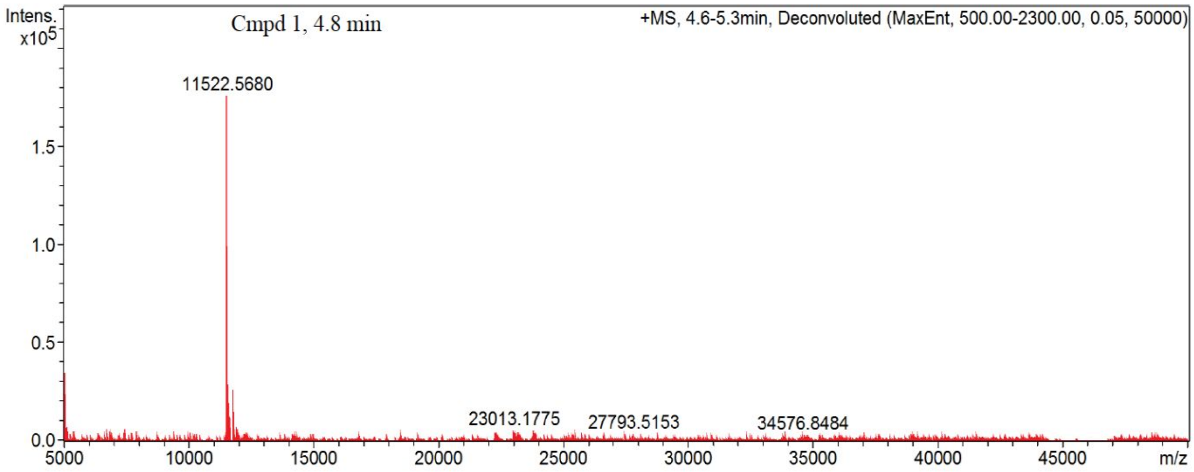

Figure S17: (A) Analytical HPLC of Z_{HER2}-HP9. (B) ESI-TOF mass spectrum of Z_{HER2}-HP9. Theoretical Mass: 11524 Da.

A

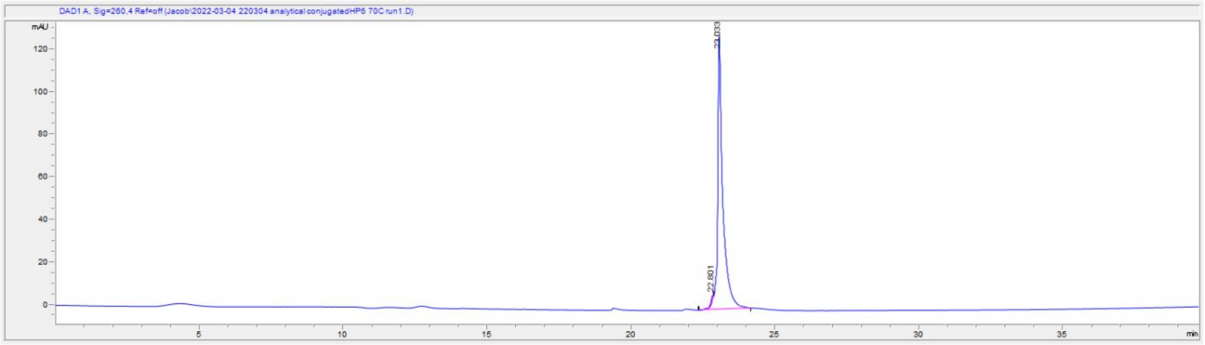

B

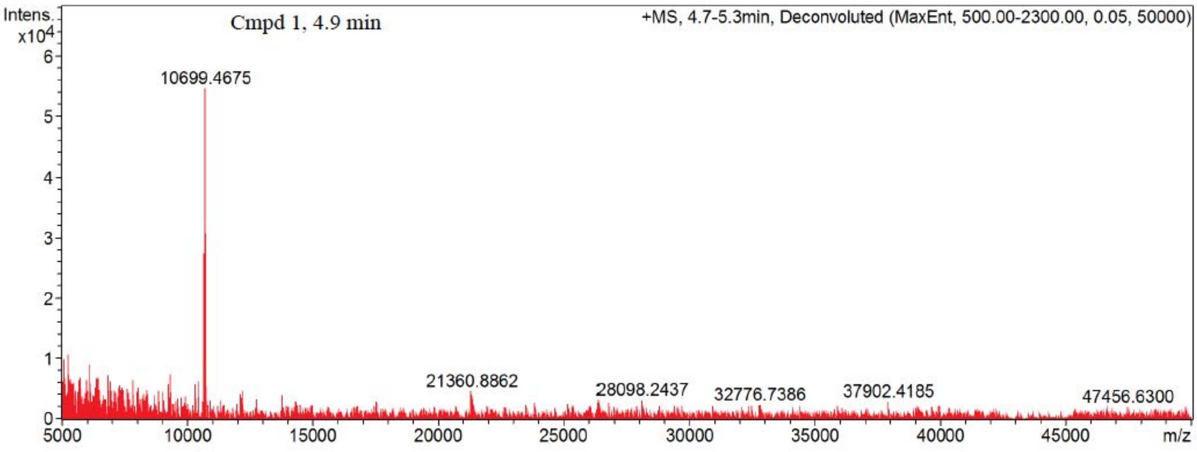

Figure S18: (A) Analytical HPLC of Z_{HER2}-HP6. (B) ESI-TOF mass spectrum of Z_{HER2}-HP6. Theoretical Mass: 10701 Da.

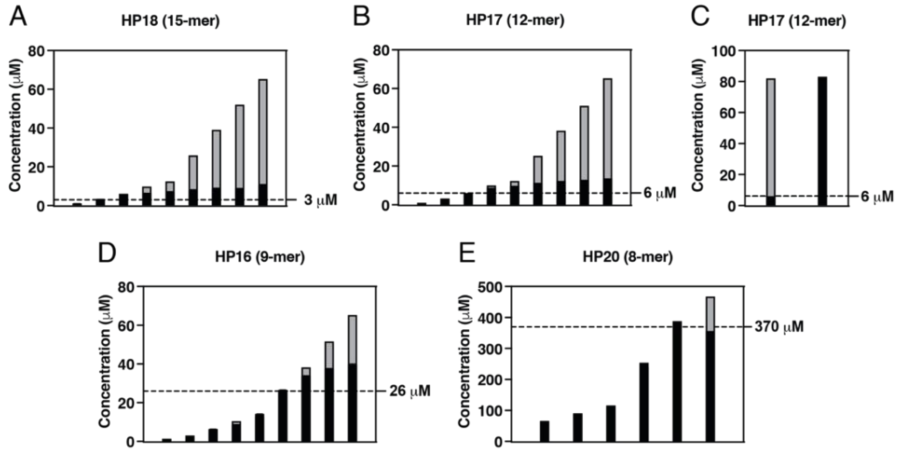

Figure S19: Estimation of solubility of secondary probes A) HP18, B) HP17, D) HP16, and E) HP20 in 0.2 M NH_4Ac, pH 5.5 at 25 ℃. Dilution series of HPLC-purified and lyophilized secondary probes (1, 3, 6, 9, 13, 26, 39, 52 and 65 μM) for HP18, HP17 and HP16, and (65, 91, 117, 240, 370 and 470 μM) for HP20 were made in 0.2 M NH_4Ac, pH 5.5. The PNA samples were heated at 95 ℃ for five minutes and concentration of the soluble fraction was determined by UV absorption at 260 nm before (grey bars), and after (black bars) an 18-hour static incubation at 25 ℃. Duplicates were prepared of all samples and the data is presented as the mean ± SD. The maximum soluble concentration of each probe, indicated in panels A-E as a dotted line, was defined as the highest concentration at which at least 95% is still in the soluble fraction after an 18-hour incubation at 25 ℃. PNA-aggregates are temperature-labile, as exemplified in panel C for 82 μM HP17. After an 18-hour incubation 25 ℃, 6 μM is left in the soluble fraction (left black bar) but heating the sample at 95 ℃ for five minutes completely redissolved HP17 aggregates into solution (right black bar).

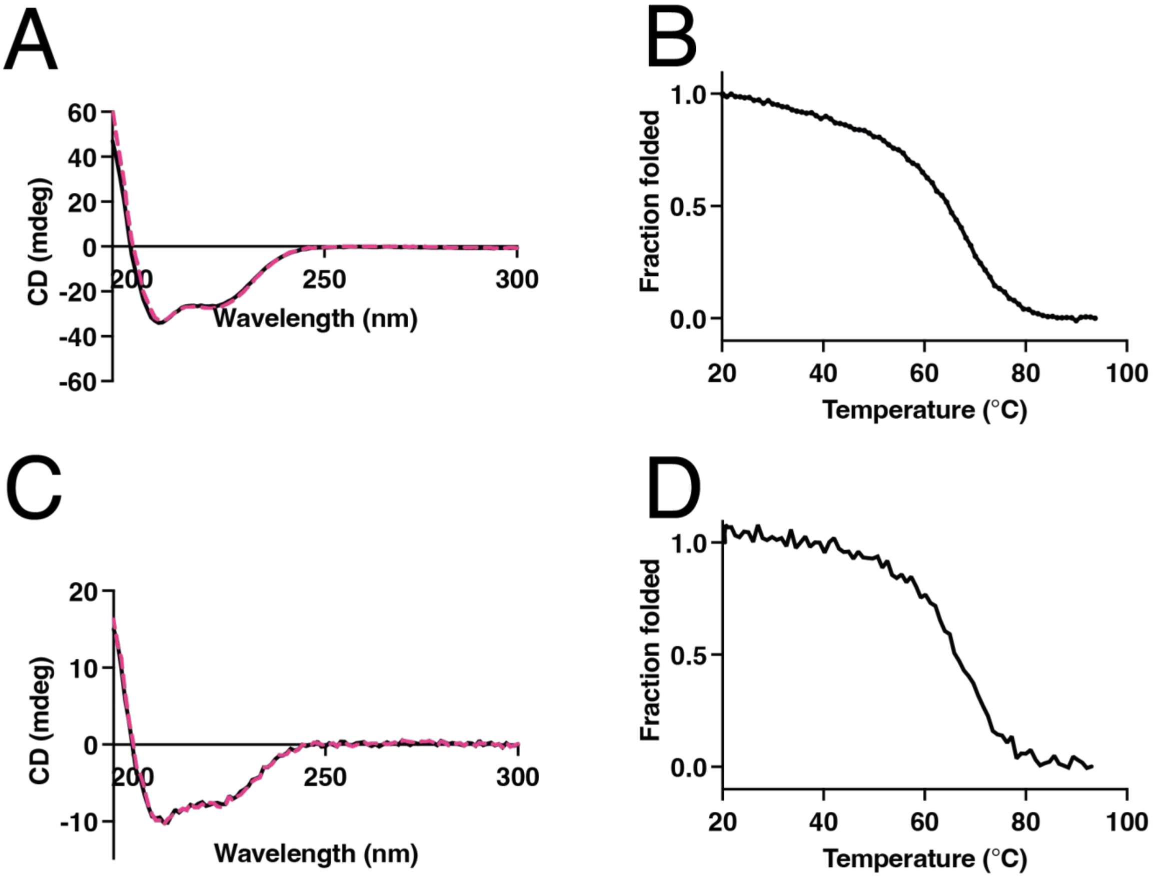

Figure S20: Circular dichroism spectra of (A) 19 µM Z-HP12 and (C) 4 µM Z-HP9 collected at 20 °C, before (black) and after (dotted pink line) thermal denaturation at 95C. Thermal denaturation of Z-HP12. The loss of secondary structure in (C) Z-HP12 and (D) Z-HP9 was followed by monitoring the CD intensity at 221 nm as a function of temperature. The data is presented here as the fraction folded protein at different temperatures. All spectra in figure S18 were recorded in 20 mM potassium buffer with 100 mM KCl; pH 7.4, and using a 2 mm quartz cuvette.

The equilibrium dissociation constant (K_D) for Z-HP12 and Z-HP9 binding to HER2-Fc was measured to 180 and 280 pM, respectively, using SPR. The binding was characterized by fast on-rates (k_a=1.0–1.2 x 10^6 M^{-1} s^{-1}) and slow off-rates (k_d=2.2–2.9 x 10^{-4} s^{-1}). The measured kinetic constants are in excellent agreement with kinetic constants measured for both the first generation, Z-HP1:HP2 (K_D= 212 pM, [1]) and second generation, Z-HP15:HP18 (K_D=276 pM, [2]), of 15-mer pretargeting agents binding to the HER2 receptor. Compared to the parental unmodified $Z_{HER2:342,}$ which has demonstrated a K_D of 22pM binding to HER2, the PNA-conjugated Affibody molecules show a 10-fold increase in K_D and are more in line with dissociation equilibrium constants (90.2–283 pM) reported earlier for $Z_{HER2:342}$ variants with short peptide extension at the C-terminal [3].

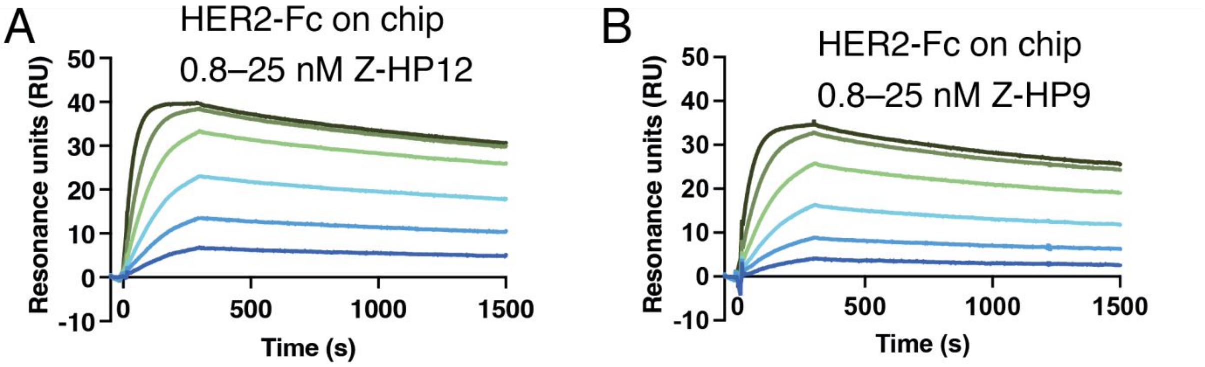

Figure S21: Representative SPR sensorgrams s depicting the interaction between immobilized HER2-Fc and Z-HP12 (A) and Z-HP9 (B). The Affibody-PNA conjugates were introduced at six different concentrations (0.8, 1.6, 3.1, 6.3, 12.5 and 25 nM) and monitored during a 20-minute dissociation period after injection for 300 seconds. The measurements are presented after double referencing (subtracting data from the reference channel and buffer injection).

Table S1: Kinetic parameters obtained from fitting the sensorgrams in figure S21 to a 1:1 binding model in the Biacore T200 software.

Protein	k_a (M^{-1} s^{-1})	k_d (s^{-1})	K_D (pM)	χ^2
Z-HP12	1.2x10^6	2.2x10^{-4}	180	0.3
Z-HP9	1.0x10^6	2.9x10^{-4}	280	0.3

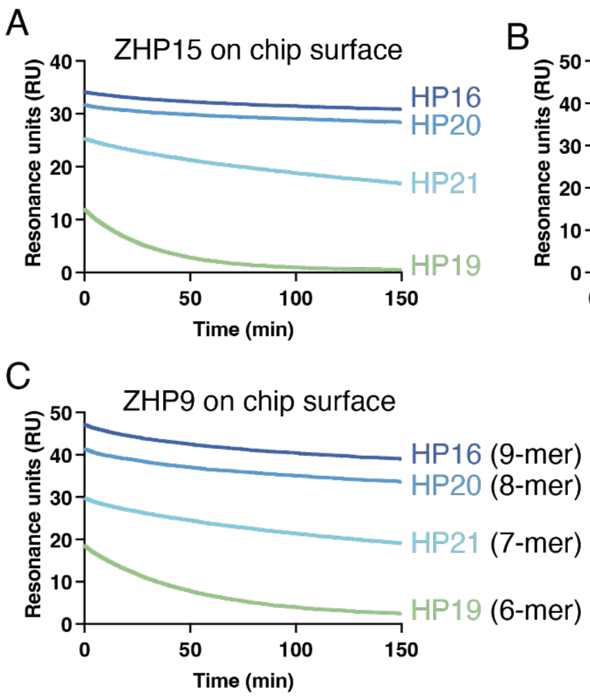

Figure S22: Representative SPR sensorgrams of the dissociation phase of HP16 (9-mer), HP20 (8-mer), HP21 (7-mer) and HP19 (6-mer) binding to immobilized primary probes Z-HP15 (A), Z-HP12 (B) and Z-HP9 (C). After injection with the secondary probe (120 nM of HP16 and HP20 or 362 nM of HP21 and HP19) the dissociation of the formed complex was followed for 150 minutes. All experiments were done in duplicates, and the data presented is double-referenced (reference channel and buffer injection are subtracted).

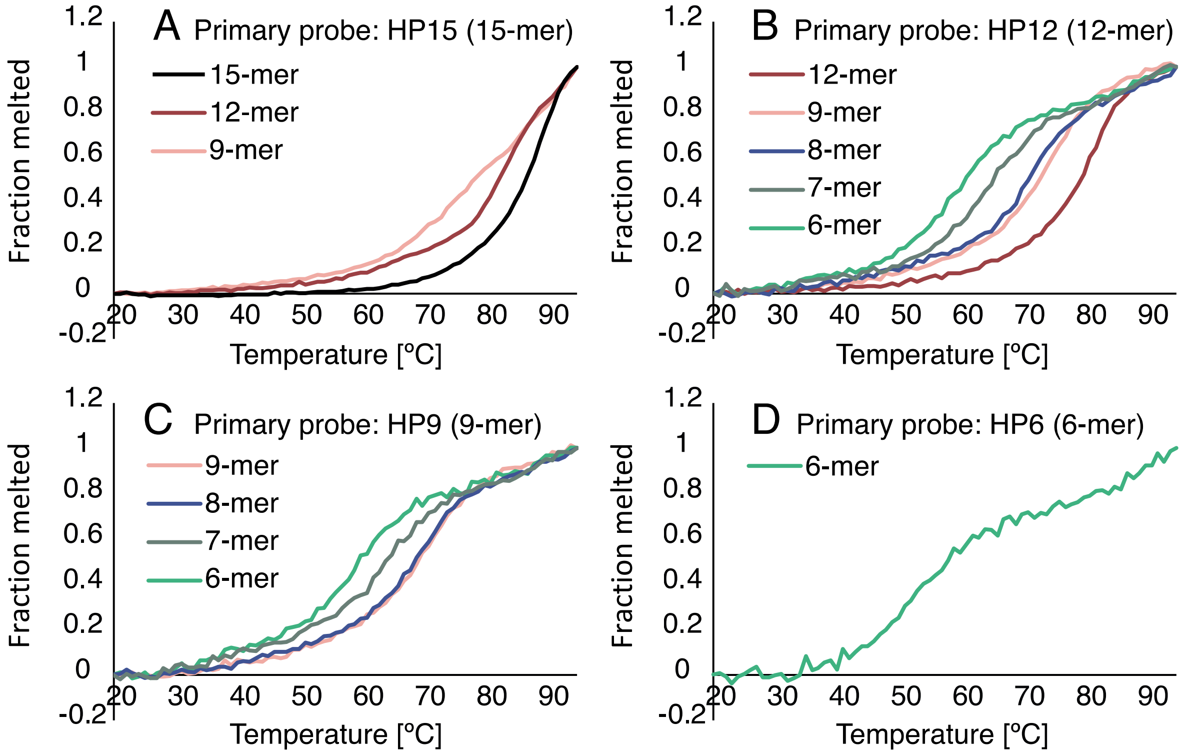

Figure S23: Normalized temperature melting profiles from 20–95 ℃ for different primary and secondary probe pairs. UV melting curves at 260 nm. (A) HP15 (15-mer) toward secondary 15-, 12- and 9-mers. (B) HP12 (12-mer) toward secondary 12-, 9-, 8-, 7- and 6-mers. (C) HP9 (9-mer) toward secondary 9-, 8-, 7- and 6-mers. (D) HP6 (6-mer) toward secondary 6-mer. Concentrations of duplexes in A–D are 5 µM in 10 mM potassium buffer with 10 mM KCl at pH 7.4.

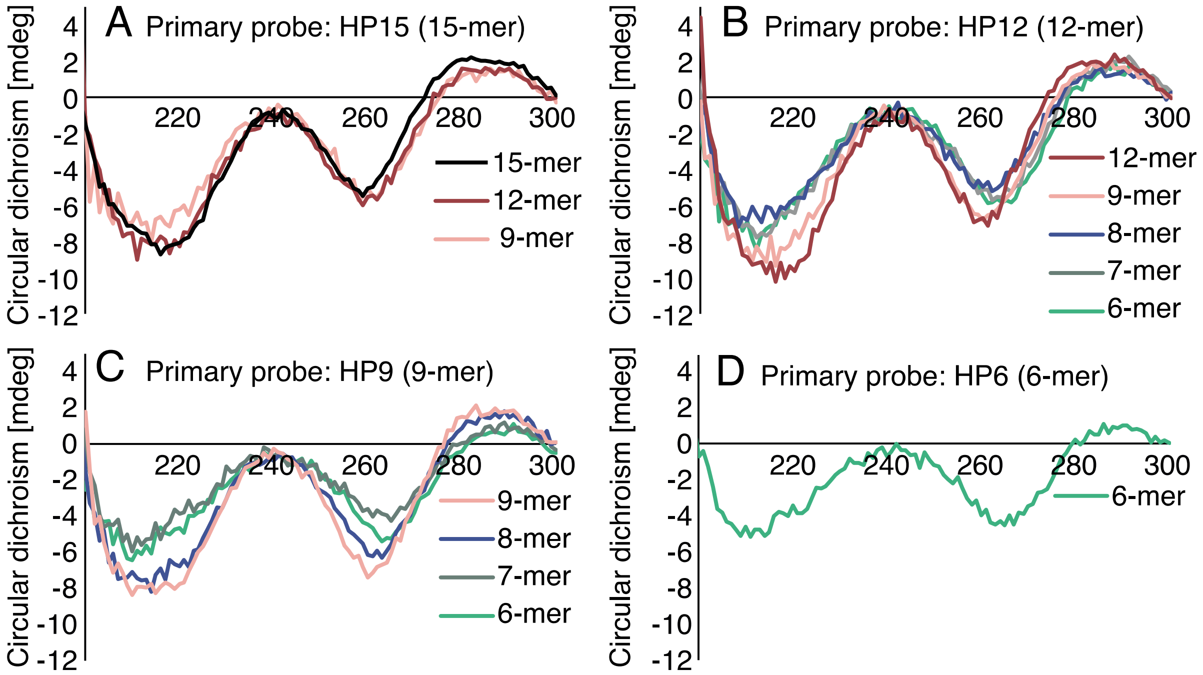

Figure S24: CD spectra of the hybridized PNA:PNA probes. (A) The 15-mer HP15 hybridized with the 15-mer HP18, the 12-mer HP17 and the 9-mer HP16. (B) The primary 12-mer probe HP12 hybridized with the 12-mer HP17, the 9-mer HP16, the 8-mer HP21, the 7-mer HP20 and the 6-mer HP19. (C) The primary 9-mer probe HP9 hybridized with the 9-mer HP16, the 8-mer HP21, the 7-mer HP20 and the 6-mer HP19. (D) The primary 6-mer probe HP6 hybridized with the secondary 6-mer probe HP19. The probes were mixed in a ratio of 1:1 at 5 µM and the samples were heated at 95°C before being allowed to cool down at room temperature.

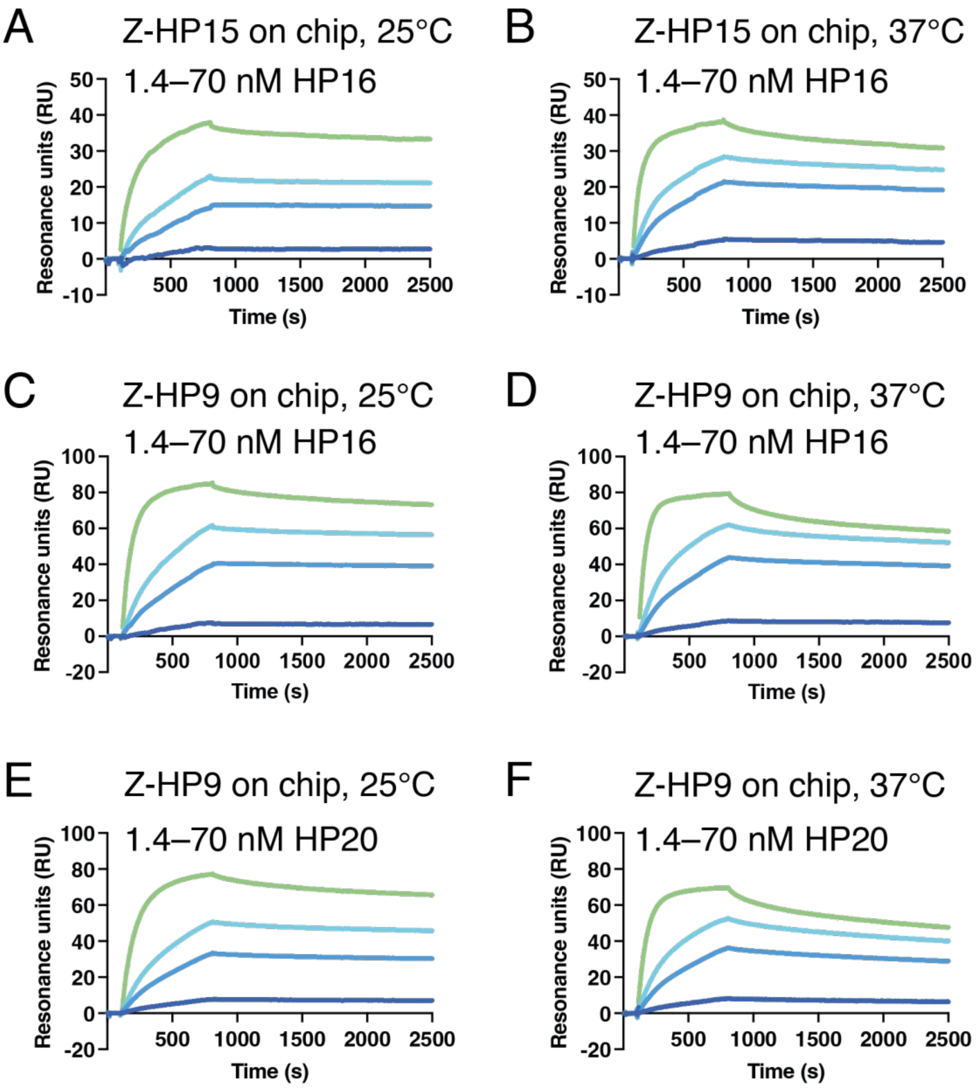

Figure S25: Sensorgrams depicting the interaction between immobilized primary probes $Z_{HER2:342}$-HP15 (A and B) and $Z_{HER:342}$-HP9 (C–F) with secondary probes, 9-mer HP16 (A–D) and 8-mer HP20 (E and F). The secondary probes were introduced at four different concentrations (1.4, 7, 14, and 70 nM) and monitored during a 30-minute dissociation period after injection for 700 seconds. Sensorgrams were recorded at two distinct temperatures, 25 ℃ (A, C, and E) and 37 ℃ (B, D, and F). All measurements were conducted in duplicate and are presented after double referencing (subtracting data from the reference channel and buffer injection).

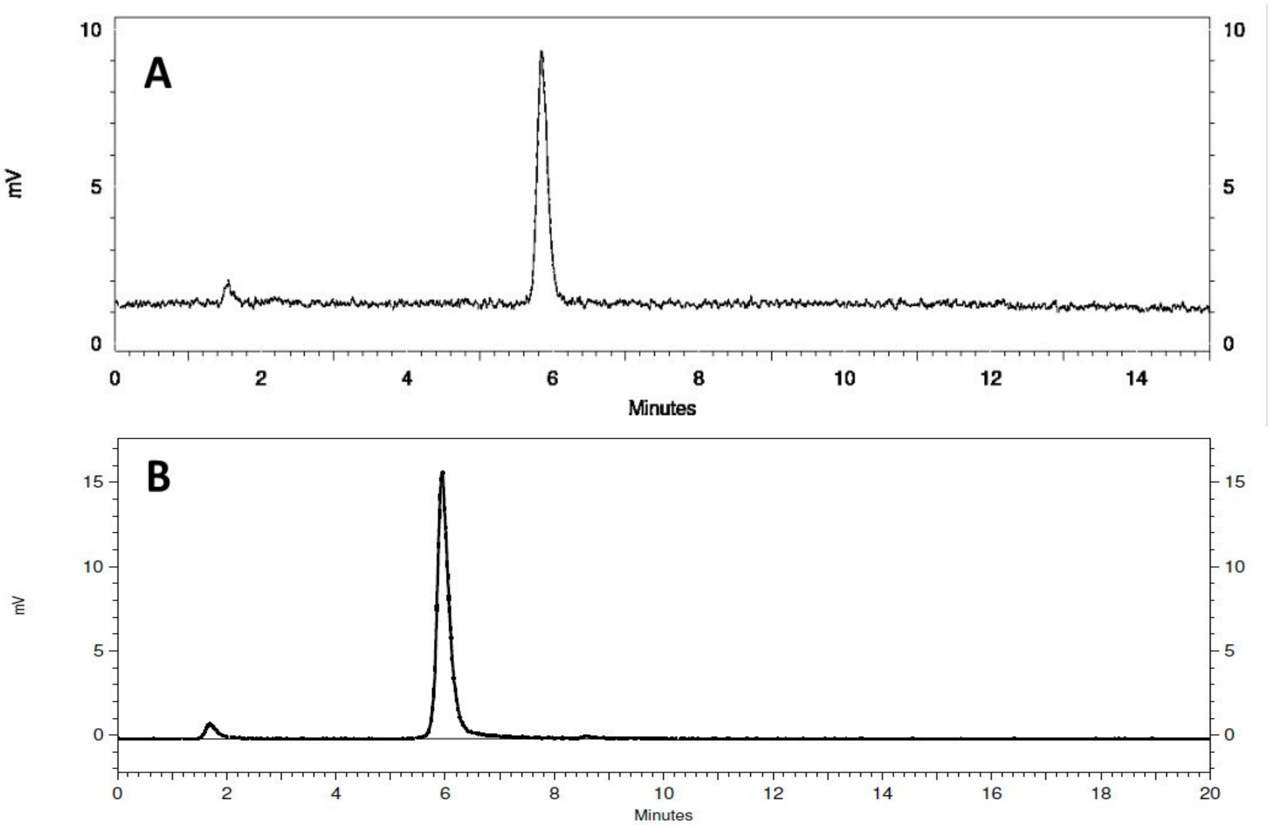

Figure S26. HPLC radiochromatograms of radiolabelled secondary agents, (A) [^{177}Lu]Lu-HP16, and (B) [^{177}Lu]Lu-HP20. The retention times are expressed in minutes.

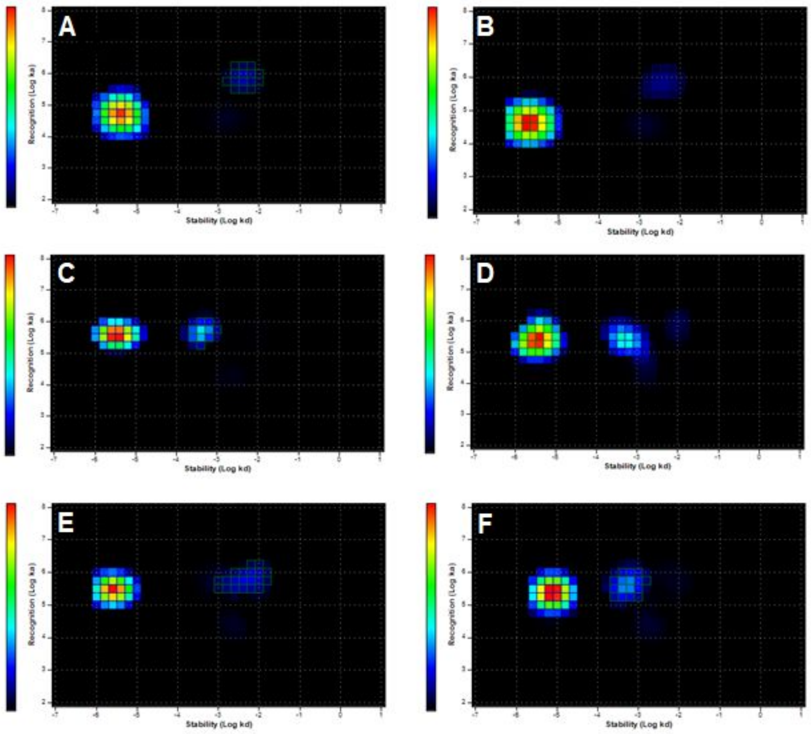

Figure S27. InteractionMap of primary agents, (A) [^{177}Lu]Lu-ZHP9 , (B) [^{177}Lu]Lu-ZHP12 , and secondary agents [^{177}Lu]Lu-HP16 pre-incubated with (C) ZHP9 and (D) ZHP12; (E) [^{177}Lu]Lu-HP20 pre-incubated with ZHP9 and (F) ZHP15 on SKOV-3 cell line. There was no binding between [^{177}Lu]Lu-HP20 pre-treated with ZHP12. Binding was measured at two different concentrations of 180, 540 pM, and 1.62 nM for primary probes (A and B), and 1 and 5 nM for secondary probes (C-F), respectively. All measurements were performed in duplicates.

Table S2. Comparative biodistribution of [^{177}Lu]Lu-HP16 and [^{177}Lu]Lu-HP20 in BALB/C nu/nu mice bearing SKOV3 xenografts at 4 h post-injection without pre-injection of a primary agent. Data are presented as an average of %ID/g ± SD, n = 4.

Organ	Uptake, %ID/g	
	[^{177}Lu]Lu-HP16	[^{177}Lu]Lu-HP20
Blood	0.02 ± 0.02	0.01 ± 0.004
Lung	0.10 ± 0.03	0.07 ± 0.01
Liver	0.13 ± 0.01	0.15 ± 0.01
Spleen	0.08 ± 0.01	0.07 ± 0.01
Kidney	4.41 ± 0.61	4.86 ± 0.85
Tumour	0.09 ± 0.01	0.11 ± 0.03
Muscle	0.05 ± 0.02*	0.02 ± 0.01*
Bone	0.04 ± 0.01	0.03 ± 0.02
GI **	0.61 ± 0.16	1.25 ± 1.06
Carcass **	1.49 ± 0.42	1.10 ± 0.34

**Significant (p<0.05) difference; **The gastrointestinal (GI) and carcass data are expressed as %ID per whole sample.*

Supplementary Material and methods

Solubility of secondary probes

HPLC-purified and freeze-dried secondary probes were resuspended in 10 mM NaAc, pH 3.5-3.6 and freeze-dried in smaller aliquots. The secondary probes were re-dissolved in 0.2 M NH_4Ac, pH 5.5, and the samples were heated at 95 °C for five minutes to ensure complete dissolvement. Dilution series of secondary probes (1, 3, 6, 9, 13, 26, 39, 52 and 65 µM) for HP18, HP17 and HP16, and (65, 91, 117, 240, 370 and 470 µM) for HP20 were made in 0.2 M NH_4Ac, pH 5.5. The samples were then left for an 18-hour static incubation at 25 C, before being centrifugated at 21,100 x g for 5 minutes on a standard benchtop centrifuge (Eppendorf). The supernatants were transferred to fresh 1.5 mL Eppendorf-tubes, and the probe concentrations were determined by measuring the UV absorption at 260 nm. All measurements were done in duplicates and are presented in figure 7 as the mean +/- SD. The maximum soluble concentration was defined as the highest concentration at which at least 95% of the original concentration was left in the supernatant after the incubation and centrifugation steps.

Biophysical characterization of PNA:PNA duplexes using CD Spectroscopy and UV melting

Circular dichroism (CD) and UV melts of PNA:PNA duplexes were measured on a CD spectrometer (Applied Photophysics, Leatherhead, UK) with Peltier temperature control and an external temperature probe. RP-HPLC purified and lyophilized PNA probes were dissolved in 10 mM phosphate buffer with 10 mM KCl at pH 7.4. Samples contained both primary and complementary secondary PNA probes at a final concentration of 5 µM for each probe. These samples were initially heated to 95°C for five minutes, allowed to cool to room temperature, and then transferred to a quartz cuvette with a 1.0 cm path length. A temperature probe was inserted into the solution through the cuvette's rubber cap.

For UV melting experiments, the temperature in the cuvette was gradually increased from 20°C to 95°C with a 1°C/min temperature gradient. Simultaneously, the UV absorbance at 260 nm was recorded as a function of temperature. Fraction melted data was calculated using the formula:

$$\text{Fraction melted} = 1 - \frac{\text{UV}_{260} - \text{UV}_{ss}}{\text{UV}_{ds} - \text{UV}_{ss}},$$

where UV_{ss} and UV_{ds} are the UV absorbances at 95°C and 20°C, respectively. The melting temperature (T_m) was determined as the maximum of the first derivative of the melting curve and was computed using the Global 3 analysis software (Applied Photophysics, Leatherhead, UK).

CD spectra in the range of 200–300 nm were recorded for all hybridized PNA:PNA pairs at 20°C before and after the UV melting experiments. All CD spectra were baseline subtracted and presented as an average of five scans.

Kinetic characterization of Z-HP12 and Z-HP9 binding to HER2 using Surface Plasmon Resonance

We utilized Surface Plasmon Resonance (SPR) on a Biacore T200 instrument (Cytiva) to examine how Z-HP12 and Z-HP9 bind to the HER2 receptor at 25°C. These experiments were conducted using dextran-coated CM5 sensor chips and PBS-T buffer at pH 7.4. Recombinant human HER2/Fc chimera (Sino Biologicals) was immobilized through a standard EDC/NHS coupling method at 610, 640 and 750 RU on a CM5 chip, and the chip surface was sealed with ethanolamine. A reference surface with no primary agent was also included. We injected various concentrations of Z-HP12 and Z-HP9 (0.8, 1.6, 3.1, 6.3, 12.5 and 25 nM) over all three chip surfaces at a flow rate of 50 µL/min. Association and dissociation times were set at 300 and 1200 seconds, respectively, with regeneration accomplished through a 30-second injection of 10 mM glycine-HCl, pH 1.5.

Data analysis was conducted using the Biacore T200 Evaluation software version 2.0 with a 1:1 binding model.

Supplementary References

[1]. Westerlund K, Honarvar H, Tolmachev V, Eriksson Karlström A. Design, Preparation, and Characterization of PNA-Based Hybridization Probes for Affibody-Molecule-Mediated Pretargeting. *Bioconjug Chem*. 2015;26(8):1724-1736. doi:10.1021/acs.bioconjchem.5b00292

[2]. Tano H, Oroujeni M, Vorobyeva A, Westerlund K, Liu Y, Xu T, Vasconcelos D, Orlova A, Karlström AE, Tolmachev V. Comparative Evaluation of Novel ^{177}Lu-Labeled PNA Probes for Affibody-Mediated PNA-Based Pretargeting. Cancers (Basel). 2021 Jan 28;13(3):500. doi: 10.3390/cancers13030500. PMID: 33525578; PMCID: PMC7865858.

[3]. Wållberg H, Orlova A, Altai M, Hosseinimehr SJ, Widström C, Malmberg J, Ståhl S, Tolmachev V. Molecular design and optimization of 99mTc-labeled recombinant affibody molecules improves their biodistribution and imaging properties. J Nucl Med. 2011 Mar;52(3):461-9. doi: 10.2967/jnumed.110.083592. Epub 2011 Feb 14. PMID: 21321280.
